# ﻿A never-ending story: updated 3D cyber-taxonomic revision of the ant genus *Zasphinctus* Wheeler (Hymenoptera, Formicidae, Dorylinae) for the Afrotropical region

**DOI:** 10.3897/zookeys.1223.131238

**Published:** 2025-01-06

**Authors:** Francisco Hita Garcia, Kiko Gómez, Roberto A. Keller, Bernhard Schurian, Evan P. Economo

**Affiliations:** 1 Center for Integrative Biodiversity Discovery, Museum für Naturkunde Berlin, Berlin, Germany; 2 Biodiversity and Biocomplexity Unit, Okinawa Institute of Science and Technology Graduate University, Onna-son, Japan; 3 Garraf, Barcelona, Spain; 4 Museu Nacional de História Natural e da Ciência and CE3C - Centre for Ecology, Evolution and Environmental Changes and CHANGE - Global Change and Sustainability Institute, Universidade de Lisboa, Lisbon, Portugal; 5 Digitisation / Technology Development, Museum für Naturkunde Berlin, Berlin, Germany

**Keywords:** 3D-model, cybertype, micro-CT, morphology, new species, taxonomy

## Abstract

The ant genus *Zasphinctus* are fascinating ants due to their distinctive morphology, ecology, and rarity. In this study, a comprehensive revision of *Zasphinctus* in the Afrotropical region is presented, through a combination of morphological examination under the light microscope and three-dimensional (3D) cyber-taxonomy based on microtomography (micro-CT). Micro-CT based 3D surface models of all species were used for virtual morphological visualisation and examination. The 3D models were virtually visualised, rotated, scaled, and dissected in order to obtain the best shape data for whole specimens or individual body parts. This approach offered a greatly improved character evaluation, allowing the development of an updated taxonomic species delimitation system for the genus. Our revision recognises eight worker-based species, of which three were previously known and five are newly described in this study. Furthermore, based on distinctive morphological differences, two species groups are also proposed. The *Z.obamai* group includes the species *Z.obamai* Hita Garcia, 2017 (Kenya), *Z.lumumbai* Hita Garcia & Gómez, **sp. nov.** (Democratic Republic of Congo), and *Z.wilsoni* Hita Garcia, 2017 (Mozambique) while the *Z.sarowiwai* group contains *Z.aprilia* Hita Garcia & Gómez, **sp. nov.** (Democratic Republic of Congo, Uganda), *Z.kouakoui* Hita Garcia & Gómez, **sp. nov.** (Ivory Coast), *Z.lolae* Hita Garcia & Gómez **sp. nov.** (Ghana), *Z.ndouri* Hita Garcia & Gómez, **sp. nov.** (Senegal), and *Z.sarowiwai* Hita Garcia, 2017 (Cameroon). All species are easily distinguishable through a comprehensive character matrix illustrated by numerous diagnostic illustrations, as well as a traditional dichotomous identification key.

## ﻿Introduction

The Afrotropical region is of crucial importance for insect biodiversity due to its unique ecosystems, variety of bioregions, as well as high species richness and endemism. The region has also been recognised as a hotspot for ant diversity (Robertson 2000; Ward 2000; Fisher 2009; Guénard et al. 2012), but recent assessments concluded that Afrotropical ant diversity might be relatively less diverse compared to other regions (Kass et al. 2022). However, such findings might be due to a lack of comprehensive sampling compared to these other regions (Jimoh et al. 2024), which is supported by recent generic revisions that underline the view that the richness of the Afrotropical ant fauna has been vastly underestimated (Hita Garcia et al. 2019; Gómez 2022).

Afrotropical ant taxonomy lags behind our advances in biogeographic regions, such as the Malagasy or Neotropical for a variety of reasons, the most important ones being the lack of modern systematic sampling for most of the region, scarcity of qualified taxonomists, and lack of funding. The majority of the most species-rich genera have never been revised and their taxonomy remains at the level of the late 19^th^ and first half of the 20^th^ centuries (Robertson 2000; Hita Garcia et al. 2013). Diverse genera that have received one or more modern taxonomic revisions on a genus level were done mostly in the 70s or 80s of the last century, such as *Tetramorium* Mayr (Bolton 1976, 1980, 1985) or *Monomorium* by Bolton (1987). However, despite being excellently treated back then, their taxonomy is already outdated due to a constant stream of newly discovered undescribed species turning up in collections. The estimated number of ant species present in the Afrotropical region is expected to at least double its current numbers (Robertson 2000).

With currently 24 valid species, the ant genus *Zasphinctus* Wheeler is a rather moderately small Old-World genus, which can be found in the Afrotropical, Indomalayan, and Australasian regions (Antmaps, Janicki et al. 2016; Bolton 2024). Most species are known from the latter (17 spp. from Australia, New Guinea, and New Caledonia), whereas there is only one known from Southeast Asia (Jaitrong et al. 2016), another from India (Sadasivan and Kripakaran 2022), and five from the Afrotropics (Hita Garcia et al. 2017a). In general, specimens of *Zasphinctus* are somewhat rarely collected and the available material is restricted to few natural history collections. This rarity seems to be due to their rather cryptic subterranean biology. Based on current and limited knowledge of Australian *Zasphinctus*, these ants are myrmecophagous and prey on larvae and adults of a variety of other ant species captured during nest raids (Wilson 1958; Brown 1975; Buschinger et al. 1989). The morphological data on cuticle thickness, buccal mouthparts, musculature, and stinger laid out in Hita Garcia et al. (2017a) also supports this predatory lifestyle for the Afrotropical fauna.

Globally, the alpha taxonomy of the genus is in moderate condition. The largest known fauna in Australia has not been revised since Brown (1975) and would likely benefit from an updated revisionary treatment. The few Southeast Asian and Indian species were described very recently (Jaitrong et al. 2016; Sadasivan and Kripakaran 2022). The Afrotropical fauna was revised in Hita Garcia et al. (2017a) with three newly described species and a newly developed worker-based species delimitation system. However, since then the material of *Zasphinctus* available for taxonomic study has greatly increased, either by recent collections, such as in Senegal or Ghana by the second author, or by older material from natural history collections that became accessible to us, in particular from the natural history museums in London and Brussels.

The field of insect taxonomy has progressed at great pace within the last two decades through the implementation of novel computational, laboratory, and analytic tools and methods, such as DNA barcoding (e.g., Hebert and Gregory 2005; Miller 2007), molecular phylogenetics (e.g., Giribet 2010; Ward 2011), quantitative morphology (e.g., Csősz and Fisher 2016), or integrative approaches combining different lines of evidence (e.g., Schlick-Steiner et al. 2010). In recent years, an additional visualisation tool has entered the taxonomic stage: interactive three-dimensional (3D) imagery through x-ray microtomography (micro-CT). It is a state-of-the-art imaging technology that generates virtual, high-resolution, and interactive 3D reconstructions of whole specimens or particular body parts, thus allowing a maximum of morphological accuracy and fidelity (Faulwetter et al. 2013; Friedrich et al. 2014).

Within the last decade micro-CT started to be used for invertebrate taxonomy of myriapods (Stoev et al. 2013; Akkari et al. 2015, 2018), spiders (Michalik and Ramírez 2013), earthworms (Fernández et al. 2014), and flatworms (Carbayo et al. 2016). Even though the potential for insect taxonomy is enormous, micro-CT has so far mostly been used for lepidopterans (e.g., Simonsen and Kitching 2014; Moraes et al. 2023; Englund et al. 2024) and ants (e.g., Fischer et al. 2016; Sarnat et al. 2016, 2019; Agavekar et al. 2017; Hita Garcia et al. 2017b, 2019; Staab et al. 2018). The use of micro-CT provides the means for non-invasive and rapid generation of practically artefact-free morphological data for visualisation in 3D (Faulwetter et al. 2013; Friedrich et al. 2014). The non-destructive nature of the technique is of critical importance for taxonomic research since it permits the use of rare material from natural history collections, even type material (Hita Garcia et al. 2017a). Moreover, a crucial advantage of using 3D models based on micro-CT data is the option to include virtual 3D cybertypes in addition to the physical types. Based on some initial papers evaluating and pioneering the idea of openly available cybertype datasets linked to the original, physical type material (Faulwetter et al. 2013; Stoev et al. 2013; Akkari et al. 2015), it has been progressively extended and applied for ant taxonomy, with presently cybertype datasets of more than 40 species (Agavekar et al. 2017; Hita Garcia et al. 2017a, 2017b, 2019; Staab et al. 2018; Sarnat et al. 2019; Sharaf et al. 2019; Gómez et al. 2022). A detailed and critical assessment of the technology and its applications for ant taxonomy was provided by Hita Garcia et al. (2017a, 2017b, 2019).

In this study, we provide an updated taxonomic revision of the ant genus *Zasphinctus* for the Afrotropical region based on the worker caste. We describe five species as new to science and redescribe the three species treated in Hita Garcia et al. (2017a). As in the latter, our taxonomic decision-making is based on a thorough investigation of all available physical worker specimens in combination with virtual examinations of 3D surface reconstructions from high-resolution microtomography (micro-CT) scanning data from several specimens per species, if available. In Hita Garcia et al. (2017a) we presented a newly developed taxonomic discrimination system consisting of a synthesis of newly discovered and traditionally used morphological characters. Herein, we test that taxonomic system by expanding the number of worker-based species from three to eight, which led us to reassess the usefulness of several characters. Consequently, we discourage or discard the usage of some characters, but at the same time we found some new characters of high diagnostic value used here for the first time in the genus. Furthermore, as in previous studies (e.g., Hita Garcia et al. 2017a, 2019; Gómez et al. 2022), we provide the complete datasets comprising the micro-CT raw data, 3D surface models, still images of shaded 3D surface models, and coloured stacked digital images, which have all been made available online as cybertype datasets for the new species.

## ﻿Materials and methods

### ﻿Abbreviations of depositories

Institutional museum collection abbreviations follow Evenhuis (2024). The material used in this study is located and/or was examined at the following institutions:

**AFRC** AfriBugs, CC., Pretoria, Gauteng, South Africa

**CASC** California Academy of Sciences, San Francisco, USA

**KGAC** Kiko Gómez Abal Collection, Barcelona, Spain

**MCZC** Museum of Comparative Zoology, Harvard University, Cambridge, USA

**MNHNC** Museu Nacional de História Natural e da Ciência, Lisbon, Portugal

**MRAC** Royal Museum for Central Africa, Tervuren, Belgium

**NHMUK** The Natural History Museum, London, UK

**NMKE** National Museums of Kenya, Nairobi, Kenya

**RBINS** Royal Belgian Institute of Natural Sciences, Brussels, Belgium

**SAMC** Iziko South African Museum, Cape Town, South Africa

**ZMHB** Museum für Naturkunde, Berlin, Germany

**ZFMK** Zoological Research Museum Alexander Koenig, Bonn, Germany

### ﻿Material examined, data availability, and terminology

We gathered almost all currently available physical material of Afrotropical *Zasphinctus* for this study from a variety of natural history collections (see above). All specimens used in this study have been databased and the data is freely accessible on AntWeb (http://www.antweb.org). Each specimen can be traced by a unique specimen identifier attached to its pin (i.e., CASENT#, KGCOL#, with # being a number).

In this study we focus exclusively on the worker caste in order to avoid confusion and contribute to two or even three parallel taxonomic systems based on worker vs queen vs male castes (Wilson 1964; Jaitrong and Yamane 2011; Hita Garcia et al. 2017a). The problematic starting situation for *Zasphinctus* prior to the previous revision was outlined in that study (Hita Garcia et al. 2017a) and the situation is still very similar with additional worker-based material from several new localities, whereas reproductives still remain scarce and completely unassociated with any workers. This situation will hopefully get resolved with future molecular sequencing, ideally some museomic approach to tackle old types and other dry mounted material in combination with multi-loci phylogenomic techniques for freshly collected specimens in ethanol.

The overall terminology for ant morphology follows Bolton (1990), Keller (2011), Borowiec (2016), and Hita Garcia et al (2017a). The terminology for the description of surface sculpturing follows Harris (1979).

### ﻿Montage images and line drawings

For the previously known species already treated in Hita Garcia et al. (2017a) we used the colour montage images from that publication, which were taken with a Leica DFC450 camera attached to a Leica M205C microscope and the Leica Application Suite v, 4.1. The raw photo stacks were then processed to single montage images with Helicon Focus v. 6. The images of the five new species were taken with a Deimos System, containing Sony ILCE 7MK III Camera, Novoflex Tube with Mitutoyo M-Plan APO 10 × and 20 × lenses and Castel-Micro Stack Rail. Illumination was done with Profoto Flash System. The raw photo stacks were then also processed with Helicon Focus v. 6. All montage images used in this publication are available online and can be seen on AntWeb. Vector illustrations were created with Adobe Illustrator v. CS 6 by tracing specimen photographs. Compound plates of still images of 3D morphology and stacked colour images were processed and compiled in Adobe Photoshop and Illustrator v. CS 6.

### ﻿Measurements and indices

**Figure 1.**
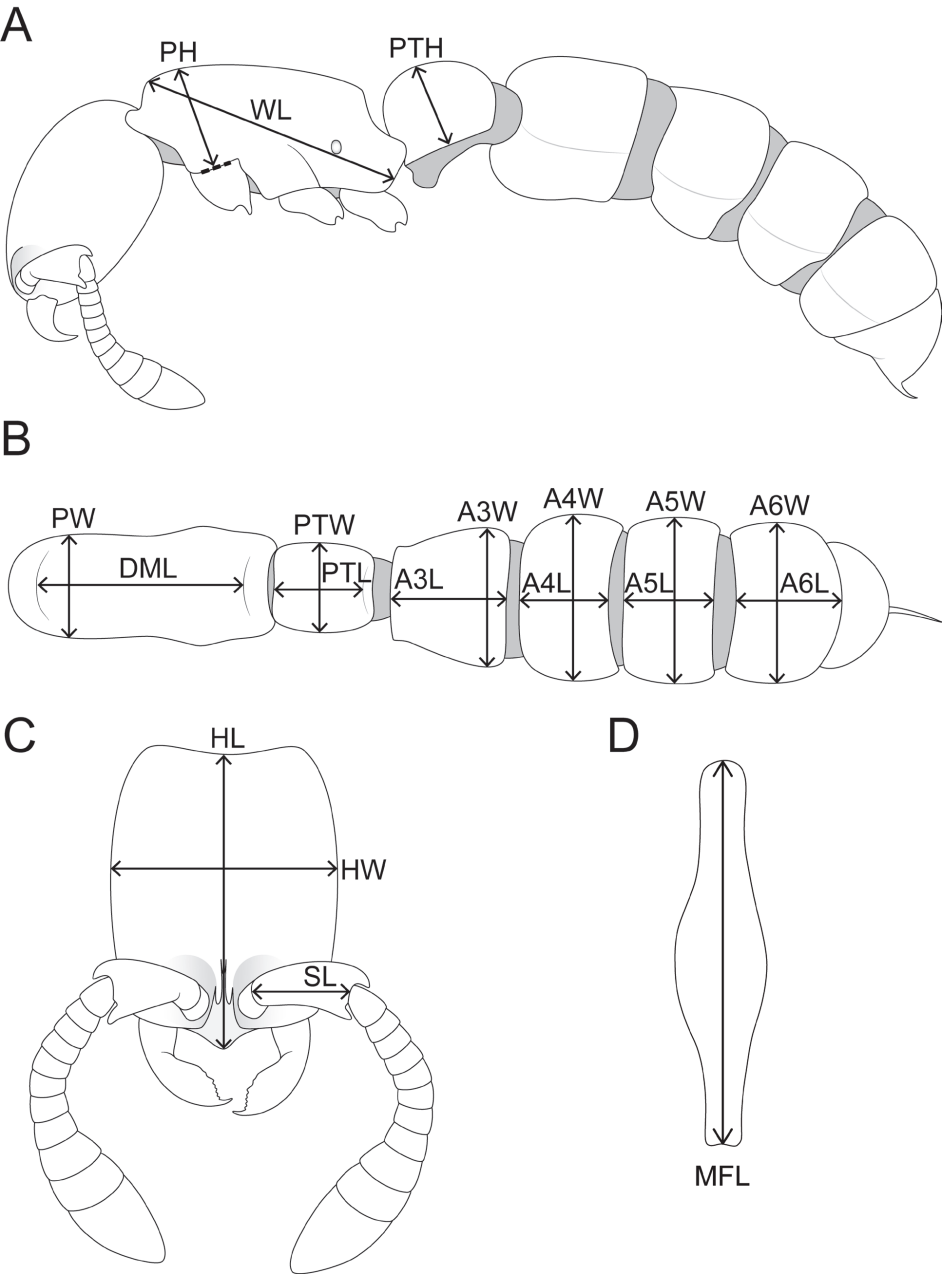
Schematic line drawings illustrating the measurements used in this study **A** body in profile with measuring lines for PH, PTH, and WL**B** mesosoma and metasoma in dorsal view with measuring lines for A3L, A3W, A4L, A4W, A5L, A5W, A6L, A6W, DML, PW, PTL, and PTW**C** head in full-face view with measuring lines for HL, HW, and SL**D** metafemur in dorsal view with measuring line for MFL.

We measured 50 workers with a Leica M165C stereo microscope equipped with an orthogonal pair of micrometres under magnifications of 80 × to 100 ×. Measurements and indices are presented as minimum and maximum values with arithmetic means in parentheses. In addition, measurements are expressed in mm to two decimal places. So far, all species of *Zasphinctus* possess eyeless workers, thus, as in Hita Garcia et al. (2017a), we omit any eye measurements and do not generate an ocular (or eye) index. Also, following that revision, we refrain from using total length since it is difficult to measure in already dry-mounted specimens that are not orientated in a straight line. The standard measurements HL and WL provide sufficient information about general body size dimensions. The following measurements and indices follow Hita Garcia et al. (2017a) (Fig. 1):

**HL** Head Length: maximum distance from the midpoint of the anterior clypeal margin or from a line spanning the anterior-most points of the frontal lobes (depending on which projects farthest forward) to the midpoint of the posterior margin of head, measured in full-face view (Fig. 1C).

**HW** Head Width: the maximum width of the head capsule, measured in full-face view (Fig. 1C).

**SL** Scape Length: the maximum straight-line length of the scape, excluding the basal constriction or the neck (Fig. 1C).

**PH** Pronotal Height: the maximum height of the pronotum in profile (Fig. 1A).

**PW** Pronotal Width: the maximum width of the pronotum in dorsal view (Fig. 1B).

**DML** Dorsal Mesosoma Length: maximum length of mesosomal dorsum from anterodorsal margin of pronotum to dorsal margin of propodeal declivity (Fig. 1B).

**WL** Weber’s Length of Mesosoma: the maximum diagonal length of the mesosoma in profile, from the angle at which the pronotum meets the cervix to the posterior basal angle of the metapleuron (Fig. 1A).

**MFL** Metafemur Length: the maximum straight-line length of the metafemur, measured in dorsal view (Fig. 1D).

**PTL** Abdominal Segment II (petiole) Length: the maximum length of abdominal segment II (petiole), measured in dorsal view (Fig. 1B).

**PTH** Abdominal Segment II (petiole) Height: the maximum height of the petiolar tergum in profile view, including laterotergite, excluding petiolar sternum (Fig. 1A).

**PTW** Abdominal Segment II (petiole) Width: the maximum width of abdominal segment II (petiole), measured in dorsal view (Fig. 1B).

**A3L** Abdominal Segment III Length: the maximum length of abdominal segment III, measured in dorsal view (Fig. 1B).

**A3W** Abdominal Segment III Width: the maximum width of abdominal segment III, measured in dorsal view (Fig. 1B).

**A4L** Abdominal Segment IV Length: the maximum length of abdominal segment IV, measured in dorsal view (Fig. 1B).

**A4W** Abdominal Segment IV Width: the maximum width of abdominal segment IV, measured in dorsal view (Fig. 1B).

**A5L** Abdominal Segment V Length: the maximum length of abdominal segment V, measured in dorsal view (Fig. 1B).

**A5W** Abdominal Segment V Width: the maximum width of abdominal segment V, measured in dorsal view (Fig. 1B).

**A6L** Abdominal Segment VI Length: the maximum length of abdominal segment VI, measured in dorsal view (Fig. 1B).

**A6W** Abdominal Segment VI Width: the maximum width of abdominal segment VI, measured in dorsal view (Fig. 1B).

**CI** Cephalic Index: HW / HL × 100

**SI** Scape Index: SL / HL × 100

**DMI** Dorsal Mesosoma Index: PW / WL × 100

**DMI2** Dorsal Mesosoma Index 2: DML / WL × 100

**LMI** Lateral Mesosoma Index: PH / WL × 100

**MFI** Metafemur Index: MFL / HW × 100

**LPI** Lateral Petiole Index: PTL / PTH × 100

**DPI** Dorsal Petiole Index: PTW / PTL × 100

**DA3I** Dorsal Abdominal Segment III Index: A3W / A3L × 100

**DA4I** Dorsal Abdominal Segment IV Index: A4W / A4L × 100

**DA5I** Dorsal Abdominal Segment V Index: A5W / A5L × 100

**DA6I** Dorsal Abdominal Segment VI Index: A6W / A6L × 100

### ﻿Micro X-ray computed tomography, virtual reconstruction, and postprocessing of data

All micro-CT scans were performed at the Okinawa Institute of Science and Technology Graduate University (OIST), Japan, using a Zeiss Xradia 510 Versa 3D X-ray microscope operated with the Zeiss Scout-and-Scan Control System software v. 11.1.6411.17883 and saved in DICOM format. The scanned specimens were left attached to their paper point, which was clamped to a holding stage. Scan settings were selected according to yield optimum scan quality and followed protocols from previous studies (Hita Garcia et al. 2017a, 2017b, 2019).

3D reconstructions of the resulting scan projection data were done with the Zeiss Scout-and-Scan Control System Reconstructor v. 11.1.6411.17883 and saved in DICOM file format. Postprocessing of DICOM raw data was performed with Amira software v. 6.3. 3D visualisations of the surface models were performed by using the ‘volren’ function. The desired volume renderings were generated by adjusting colour space range to a minimum so that the exterior surface of specimens remained visible at the highest available quality. The 3D models were then exported in PLY format to be uploaded to the online 3D model platform Sketchfab (https://sketchfab.com) and for virtual examination with Meshlab (Cignoni et al. 2008; https://www.meshlab.net). Images of shaded surface display volume renderings were made with the built-in “snapshot” function at the highest achievable resolution (1918 by 934 pixels).

### ﻿Character recognition and virtual dissections

Following Hita Garcia et al. (2017a), we virtually examined external morphology of the treated species in addition to the traditional morphological examination of the physical specimens under a light microscope with magnifications up to 100 ×. Initially we used the 24 characters of high diagnostic value identified in Hita Garcia et al. (2017a) as a foundation to examine the morphology of all material. This was also a unique opportunity to test the robustness of that species delimitation system considering that the total number of specimens, locations, and species got significantly increased since its publication in 2017.

**Figure 2.**
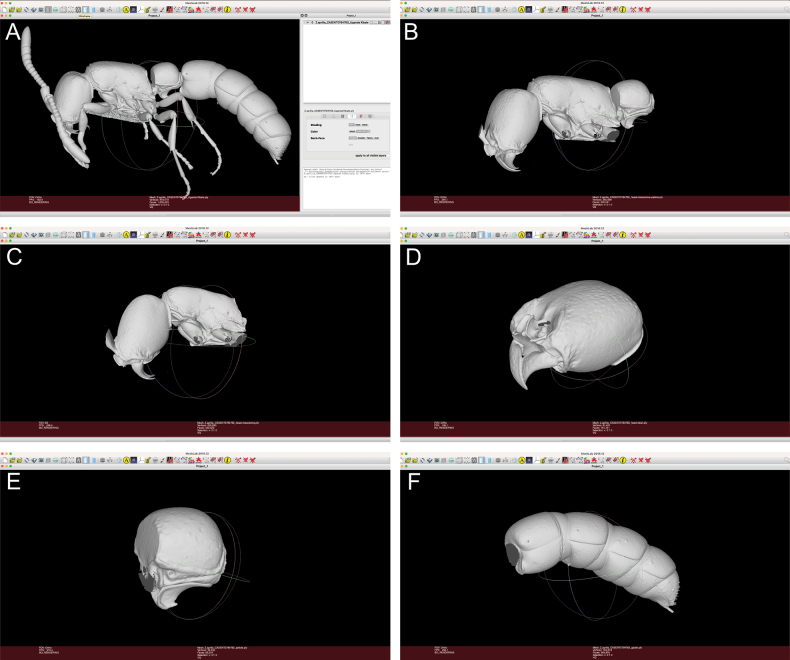
Representative screenshots of virtual dissection of exemplary 3D model, *Zasphinctusaprilia* sp. nov. holotype (CASENT0764763), in the software Meshlab **A** full body uncut **B** body after removal of antennae, legs, and AS III-VII **C** head and mesosoma after removal of remainder of body **D** head without antennae isolated **E** AS II (petiole) in isolated **F** AS III-VII isolated.

Meshlab allowed us a quick and efficient virtual examination and manipulation of all 3D models through rotation, scaling, dissection, and comparisons of entire specimens or particular body parts (Fig. 2). Every model was manipulated to show the desired angle in order to obtain a perfect view of the targeted morphological character. Once that was achieved, we took a still image with the "snapshot" function for further analysis and comparison among specimens and species. We took at least 21 still images of key diagnostic characters per species (one or two specimens per species) totalling more than 200 individual images used for illustrative purposes.

### ﻿Cybertypes

The Cybertype datasets of the five new species provided in this study consist of the original micro-CT volumetric datasets (in DICOM format), 3D surface models (in PLY formats), still images of shaded 3D surface models, and all stacked digital colour images for each species. All cybertype datasets of the species described herein have been archived and are freely available from the Zenodo Digital Repository (https://doi.org/10.5281/zenodo.12593275). In addition to the cybertype data on Zenodo, we also provide freely accessible 3D surface models of all treated species on Sketchfab (https://skfb.ly/oXnuw). The cybertype datasets of the three previously described species were already published in Hita Garcia et al. (2017a) and are available online from the Dryad Digital Repository (Hita Garcia et al. 2017c). It should be noted that our cybertype approach has changed since the first revision of *Zasphinctus* (Hita Garcia et al. 2017a). We no longer include 3D PDFs or 3D rotation video files in order to avoid too much data redundancy. The 3D models in PLY format we provide show the same (and more) visual data and offer much more usability since they can be freely downloaded and used with numerous types of software for multiple applications in 3D.

### ﻿Species concept and species delimitation

Due to logistical and practical reasons this study does not include molecular data, even though we would have preferred that. The number of specimens available was rather low for almost all species. In addition, almost all the material used was on loan from several natural history museums or private collections that would not permit any destructive DNA extraction. Moreover, despite the possibilities to non-destructively extract DNA, many specimens of *Zasphinctus* are comparatively old and delicate, thus not easy to process in such a way without potentially harming pilosity, cuticle, or breaking of individual body parts. As a consequence, this taxonomic revision had to be based on external morphology alone.

The species delimitations presented here are based on detailed morphological examinations of the worker caste and the identification of discrete character sets for each taxonomic entity proposed as species. Furthermore, we considered habitat, microhabitat, elevation, and distributional data as additional evidence. We follow the Unified Species Concept of De Queiroz (2007) that defines a species as a separately evolving metapopulation lineage. In this framework, criteria laid out in other species concepts, such as the biological species concept and the morphological species concept, are integrated as independent lines of evidence reflecting progress through stages of the speciation process. The discrete gaps observed in morphological and distributional characteristics of *Zasphinctus* species are considered as evidence for long-standing divergence among such separately evolving lineages. Moreover, unlike many other groups of ants, every single *Zasphinctus* species delineated here is directly recognisable on the basis of numerous morphological differences and has its unique set of characters, which strongly support our species hypotheses.

## ﻿Results

### ﻿Synoptic list of *Zasphinctus* species in the Afrotropics

Species known from workers and treated in this study:

***Zasphinctusobamai* group**:

*Zasphinctuslumumbai* Hita Garcia & Gómez, sp. nov. [D.R. Congo]

*Zasphinctusobamai* Hita Garcia, 2017 [Kenya]

*Zasphinctuswilsoni* Hita Garcia, 2017 [Mozambique]

***Zasphinctussarowiwai* group**:

*Zasphinctusaprilia* Hita Garcia & Gómez, sp. nov. [D.R. Congo, Uganda]

*Zasphinctuskouakoui* Hita Garcia & Gómez, sp. nov. [Ivory Coast]

*Zasphinctuslolae* Hita Garcia & Gómez, sp. nov. [Ghana]

*Zasphinctusndouri* Hita Garcia & Gómez, sp. nov. [Senegal]

*Zasphinctussarowiwai* Hita Garcia, 2017 [Cameroon]

Species only known from males and excluded from this study:

*Zasphinctuschariensis* Santschi, 1915 [Chad]

*Zasphinctusrufiventris* Santschi, 1915 [Benin, Mali]

### ﻿Distribution of species

**Figure 3.**
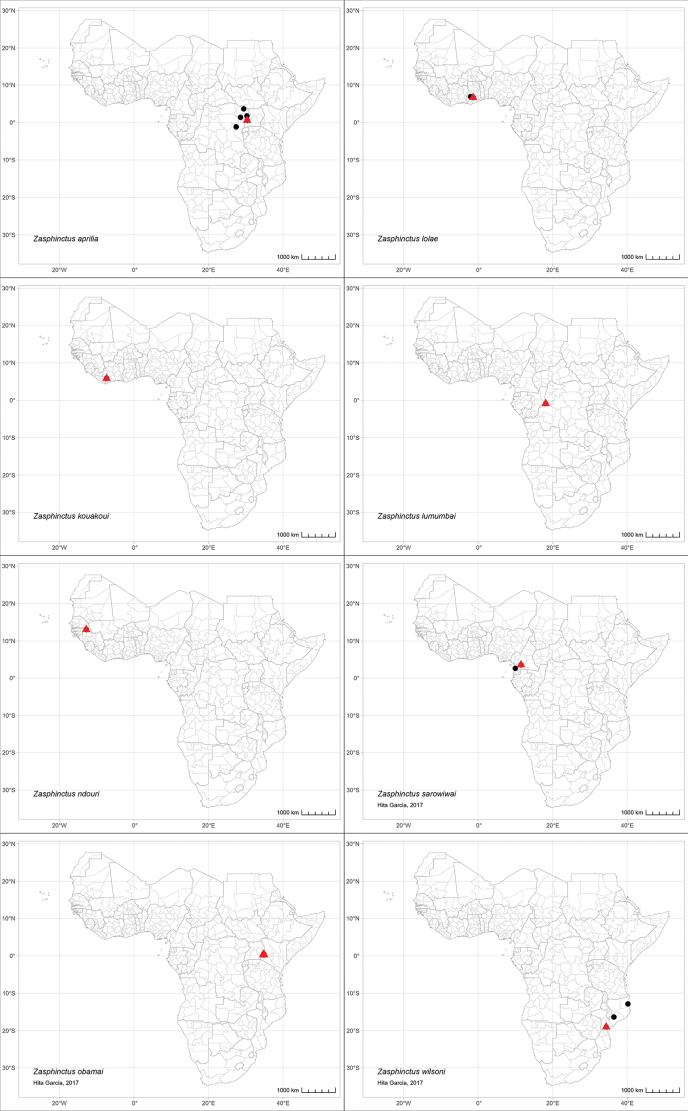
Maps showing the known distribution ranges of Afrotropical *Zasphinctus* species.

Despite that the genus is rather rarely collected compared to most other ant genera in the region, we were able to examine and revise the taxonomy of approximately 70 worker specimens that covered a wide area ranging from Senegal in the west to Kenya and Mozambique in the east and southeast, with most species and material being from Equatorial Africa. The distribution ranges of the species treated herein can be seen in Fig. 3.

*Zasphinctuschariensis* and *Z.rufiventris* are only known from males. Due to the non-existence of a taxonomic system for the male caste it is highly doubtful that males sampled throughout the region have been or can be correctly identified. As a consequence, we only list the type locality countries for these two species and strongly recommend treating all other listings encountered in online databases as highly dubious or erroneous (Antmaps; AntWeb; AntWiki, https://antwiki.org).

### ﻿Diagnostic treatment

The recent revision of Hita Garcia et al. (2017a) was based on a thorough examination of external morphology, both physically under the light microscope and virtually on the computer screen in 3D, which led to a novel way to evaluate characters and character states for species level taxonomy. Following the same approach, we used that taxonomic diagnostic system as a foundation for this study by examining the 24 diagnostic characters outlined in Hita Garcia et al. (2017a) for all our material. As a consequence, we had to exclude four characters from the list due to limited taxonomic usefulness or noticeable difficulty to visualise and apply. In particular we chose to omit the shape of the antennal scapes, the parafrontal ridges, the occiput in ventral view, and the subpetiolar process in ventral view. By contrast, we included six newly applied characters, such as the shape of the head in dorsal view, the torular-posttorular complex in profile, fusion of vertex and occiput, the postgenal sulcus running from the hypostoma to the postgenal bridge, the posterodorsal margin of the mesosoma separating the dorsum from the propodeal declivity, and surface sculpture on cephalic dorsum and genae. The complete list of diagnostic characters used for our newly developed species delimitation system, as well as the ones excluded, can be seen in Table 1.

**Table 1.** List of all important characters examined in the previous study (Hita Garcia et al. 2017a) and used as foundation for this revision, with assessment of diagnostic potential and information on usage in this study (characters marked with * were used for species delimitations in both revisions; characters marked with ± are newly used here).

Characters examined	Diagnostic assessment and usage
**Head characters**	
Shape of head in full–face view ±	high, **newly** used in this study
Shape of head in profile *	high, used in this study
Shape of mandibles	none, no significant interspecific variation observed, not used in this study
Mandibular dentition	none, no significant interspecific variation observed, not used in this study
Shape of clypeus	low, no significant interspecific variation observed, not used in this study
Presence of median clypeal tooth *	high, used in this study
Cuticular apron of clypeus	none, no significant interspecific variation observed, not used in this study
Torular–posttorular complex in dorsal view *	high, used in this study
Torular–posttorular complex in profile ±	high, **newly** used in this study
Antennal bulbus	none, no significant interspecific variation observed, not used in this study
Antennal pedicel and funiculus	none, no significant interspecific variation observed, not used in this study
Anterior tentorial pits	none, no significant interspecific variation observed, not used in this study
Eyes	none, absent in the worker caste
Vertex in posterodorsal and posterior view *	high, used in this study
Occipital margin in posterodorsal view *	high, used in this study
Fusion of vertex and occiput in posterior view ±	high, **newly** used in this study
Occipital margin in posteroventral view *	high, used in this study
Hypostoma *	high, used in this study
Postgenal sulcus running through postgenal bridge ±	high, **newly** used in this study
Mouthparts (maxillae, labium, labrum)	unclear, described in open condition for *Z.lolae*, but needs further investigation with better preserved alcohol material for μCT scanning
Tentorium (internal)	unclear, tentatively examined in Hita Garcia et al. (2017a) and appears species–specific, but needs further investigation
**Mesosoma characters**	
Mesosoma in profile *	high, used in this study
Endosternum (internal)	unclear, tentatively examined in this study and appears species–specific, but needs further investigation with better preserved alcohol material for μCT scanning
Transverse mesopleural groove	moderately variable among species, not used in this study
Propleuron	none, no significant interspecific variation observed, not used in this study
Pleural endophragmal pit *	high, used in this study
Mesopleuron	moderately variable among species, not used in this study
Metapleuron	low, no significant interspecific variation observed, not used in this study
Mesosoma dorsal *	high, used in this study
Posterodorsal margin of mesosoma ±	high, **newly** used in this study
Probasitarsus	low, no significant interspecific variation observed, not used in this study
Calcar of strigil	low, no significant interspecific variation observed, not used in this study
**Metasoma characters**	
Levator of petiole	unclear, not examined in this study, very difficult to virtually dissect
Petiolar tergum in profile *	high, used in this study
Laterotergites	low, no significant interspecific variation observed, not used in this study
Subpetiolar process of petiole (AS II) in profile * *	high, used in this study
Petiolar (AS II) tergum in dorsal view *	high, used in this study
Disc of petiole (AS II)	none, no significant interspecific variation observed, not used in this study
Helcium	unclear, not examined in this study, very difficult to virtually dissect in some specimens
Abdominal segment III in dorsal view *	high, used in this study
Abdominal segment III in ventral view *	high, used in this study
Posterior end of abdominal segment III in ventral view *	high, used in this study
Prora in anteroventral view *	high, used in this study
Abdominal segment IV in dorsal view	moderate, relatively variable within species, not used in this study
Abdominal segment IV in ventral view	moderate, relatively variable within species, not used in this study
Abdominal segment V in dorsal view	low, no significant interspecific variation observed, not used in this study
Abdominal segment V in ventral view	low, no significant interspecific variation observed, not used in this study
Abdominal segment VI in dorsal view *	high, used in this study
Abdominal segment VI in ventral view	high, not used in this study
Girdling constrictions abdominal segments IV, V, VI *	high, used in this study
Pygidium	low, no significant interspecific variation observed, not used in this study
Hypopygidium	high, not used in this study
Spiracles abdominal segments II–VII	none, no significant interspecific variation observed, not used in this study
General surface sculpture *	high, used in this study
Cuticle thickness (internal)	unclear, examined in Hita Garcia et al. (2017a) but needs further investigation with more specimens
**Setation characters**	
Pilosity and pubescence	moderate, but not used in this study due to varying degrees of preservation in many specimens and some observable intraspecific variability
**Characters used in Hita Garcia et al (2017a) but excluded here**	
Antennal scapes	moderate, but not used in this study due to difficulty in precisely measuring the scape
Parafrontal ridges	high, but not used in this study due observed intraspecific variation and difficulty in describing shape and structure
Occiput in ventral view	high, but not used in this study due to difficulty to virtually dissect in some specimens
Subpetiolar process (AS II) in ventral view	high, but not used in this study due to difficulty to virtually dissect in some specimens

**Figure 4.**
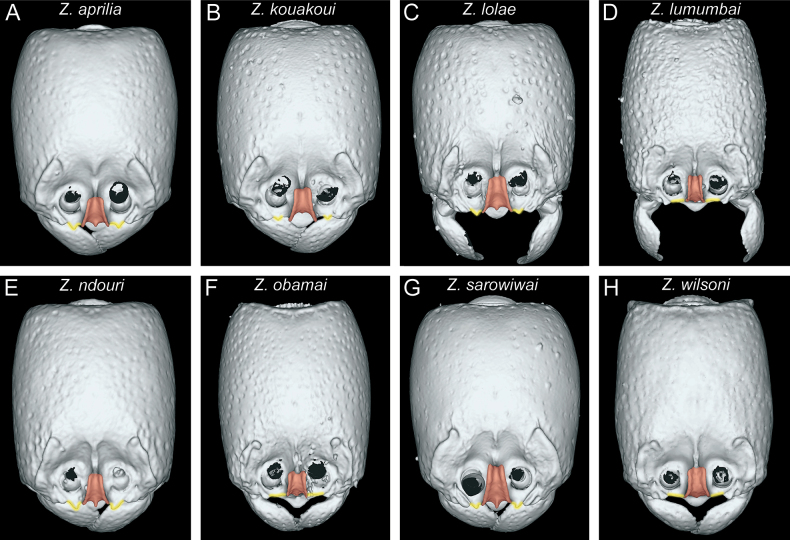
Diagnostic plate showing still images from surface volume renderings of the head in full-face view (the remainder of the body virtually removed) (torular-posttorular complex in semi-transparent red; anterior projections of parafrontal ridges in semi-transparent yellow) **A***Z.aprilia* sp. nov. holotype (CASENT0764763) **B***Z.kouakoui* sp. nov. paratype (CASENT0764653) **C***Z.lolae* sp. nov. holotype (KGCOL02270) **D***Z.lumumbai* sp. nov. holotype (MRACFOR0010007) **E***Z.ndouri* sp. nov. holotype (KGCOL01883) **F***Z.obamai* Hita Garcia, 2017 holotype (CASENT0764125) **G***Z.sarowiwai* Hita Garcia, 2017 holotype (CASENT0764654) **H***Z.wilsoni* Hita Garcia, 2017 holotype (MCZ-ENT00512764).

**Figure 5.**
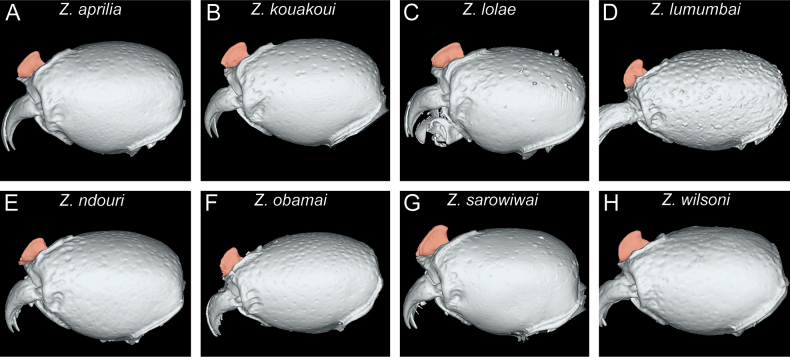
Diagnostic plate showing still images from surface volume renderings of the head in profile view (the remainder of the body virtually removed) (torular-posttorular complex in semi-transparent red) **A***Z.aprilia* sp. nov. holotype (CASENT0764763) **B***Z.kouakoui* sp. nov. paratype (CASENT0764653) **C***Z.lolae* sp. nov. paratype (CASENT0764651) **D***Z.lumumbai* sp. nov. holotype (KGCOL02270) **E***Z.ndouri* sp. nov. holotype (KGCOL01883) **F***Z.obamai* Hita Garcia, 2017 holotype (CASENT0764125) **G***Z.sarowiwai* Hita Garcia, 2017 holotype (CASENT0764654) **H***Z.wilsoni* Hita Garcia, 2017 holotype (MCZ-ENT00512764).

**Figure 6.**
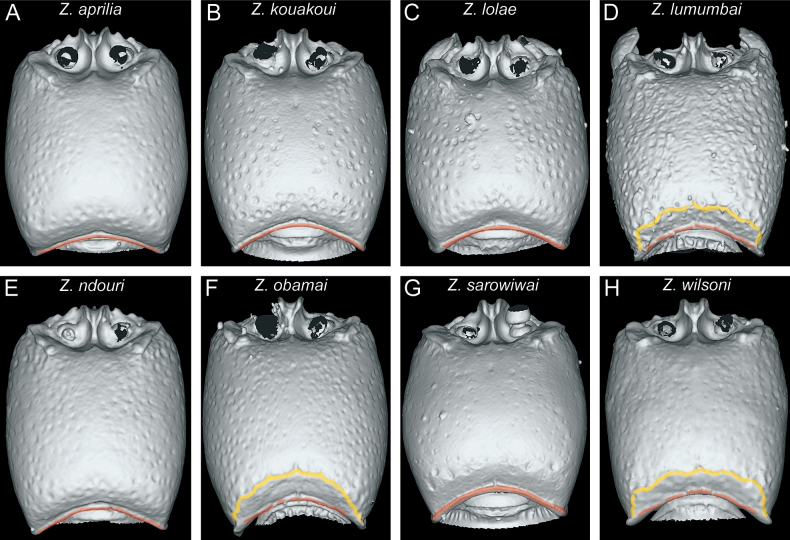
Diagnostic plate showing still images from surface volume renderings of the head in posterodorsal view (the remainder of the body virtually removed) (occipital margin in semi-transparent red; vertexal margin in semi-transparent yellow) **A***Z.aprilia* sp. nov. holotype (CASENT0764763) **B***Z.kouakoui* sp. nov. paratype (CASENT0764653) **C***Z.lolae* sp. nov. holotype (KGCOL02270) **D***Z.lumumbai* sp. nov. holotype (MRACFOR0010007) **E***Z.ndouri* sp. nov. holotype (KGCOL01883) **F***Z.obamai* Hita Garcia, 2017 holotype (CASENT0764125) **G***Z.sarowiwai* Hita Garcia, 2017 holotype (CASENT0764654) **H***Z.wilsoni* Hita Garcia, 2017 holotype (MCZ-ENT00512764).

**Figure 7.**
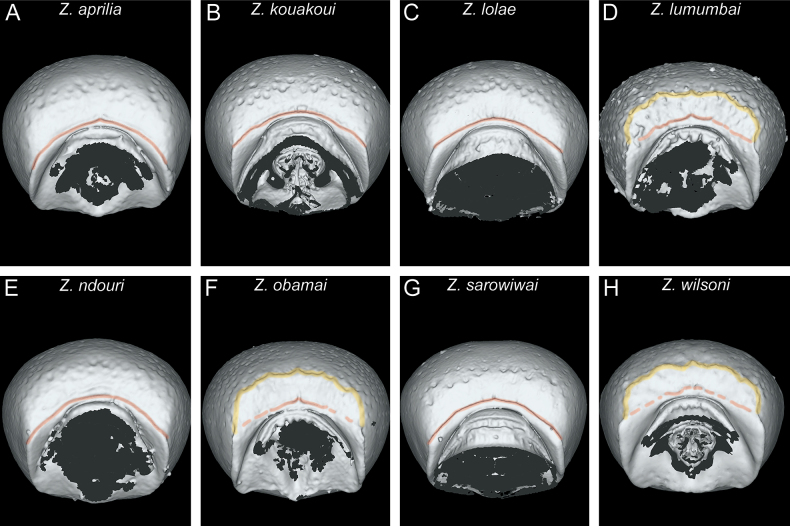
Diagnostic plate showing still images from surface volume renderings of the head in posterior view (the remainder of the body virtually removed) (occipital margin in semi-transparent red; vertexal margin in semi-transparent yellow) **A***Z.aprilia* sp. nov. holotype (CASENT0764763) **B***Z.kouakoui* sp. nov. paratype (CASENT0764653) **C***Z.lolae* sp. nov. holotype (KGCOL02270) **D***Z.lumumbai* sp. nov. holotype (MRACFOR0010007) **E***Z.ndouri* sp. nov. holotype (KGCOL01883) **F***Z.obamai* Hita Garcia, 2017 holotype (CASENT0764125) **G***Z.sarowiwai* Hita Garcia, 2017 holotype (CASENT0764654) **H***Z.wilsoni* Hita Garcia, 2017 holotype (MCZ-ENT00512764).

**Figure 8.**
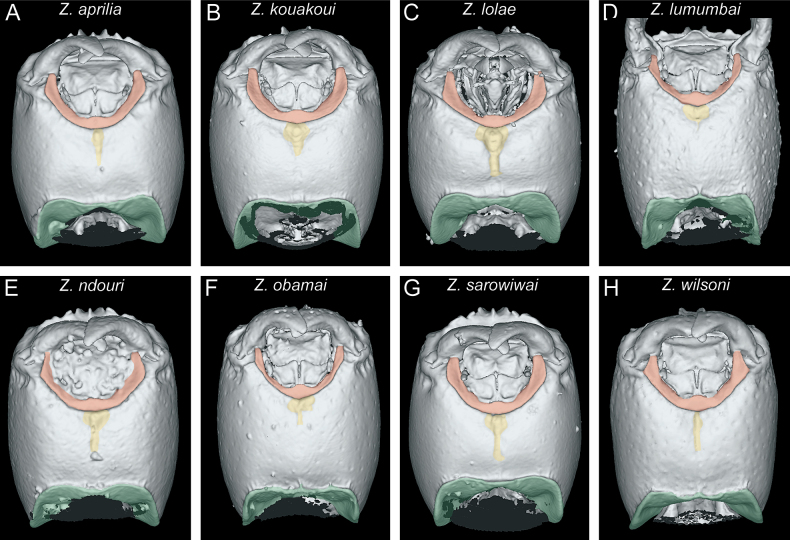
Diagnostic plate showing still images from surface volume renderings of the head in ventral view (the remainder of the body virtually removed) (hypostoma in semi-transparent red; postgenal sulcus in semi-transparent yellow; postoccipital carina in semi-transparent green) **A***Z.aprilia* sp. nov. holotype (CASENT0764763) **B***Z.kouakoui* sp. nov. paratype (CASENT0764653) **C***Z.lolae* sp. nov. paratype (CASENT0764651) **D***Z.lumumbai* sp. nov. holotype (MRACFOR0010007) **E***Z.ndouri* sp. nov. holotype (KGCOL01883) **F***Z.obamai* Hita Garcia, 2017 holotype (CASENT0764125) **G***Z.sarowiwai* Hita Garcia, 2017 paratype (CASENT0764650) **H***Z.wilsoni* Hita Garcia, 2017 holotype (MCZ-ENT00512764).

**Figure 9.**
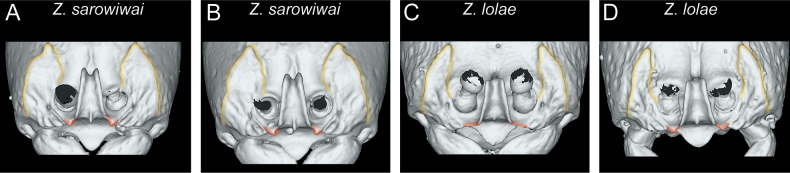
Diagnostic plate showing still images from surface volume renderings of the head in anterodorsal view focusing on the torular-posttorular complex (parafrontal ridges in semi-transparent yellow; anterior margin of parafrontal ridges in semi-transparent red) **A***Z.sarowiwai* (CASENT0764654) **B***Z.sarowiwai* (CASENT0764650) **C***Z.lolae* sp. nov. (CASENT0764651) **D***Z.lolae* sp. nov. (KGCOL02270).

**Figure 10.**
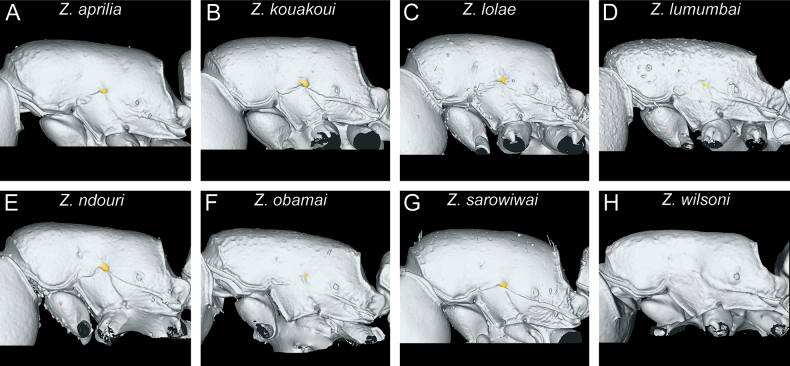
Diagnostic plate showing still images from surface volume renderings of the mesosoma in profile view (the remainder of the body virtually removed) (pleural endophragmal pit concavity in semi-transparent yellow) **A***Z.aprilia* sp. nov. holotype (CASENT0764763) **B***Z.kouakoui* sp. nov. paratype (CASENT0764653) **C***Z.lolae* sp. nov. holotype (KGCOL02270) **D***Z.lumumbai* sp. nov. holotype (MRACFOR0010007) **E***Z.ndouri* sp. nov. holotype (KGCOL01883) **F***Z.obamai* Hita Garcia, 2017 holotype (CASENT0764125) **G***Z.sarowiwai* Hita Garcia, 2017 paratype (CASENT0764650) **H***Z.wilsoni* Hita Garcia, 2017 holotype (MCZ-ENT00512764).

**Figure 11.**
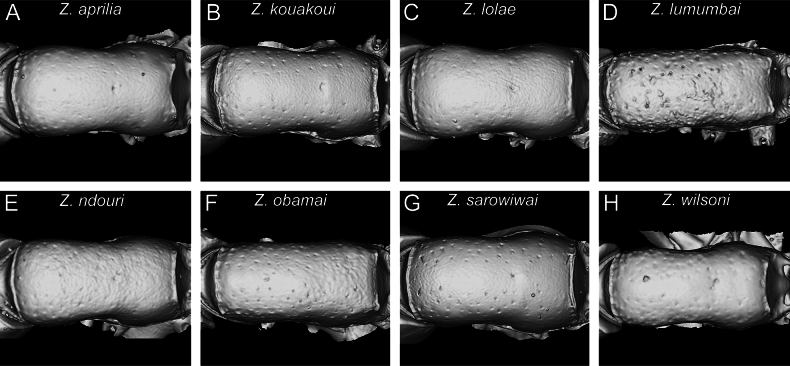
Diagnostic plate showing still images from surface volume renderings of the mesosoma in dorsal view (the remainder of the body virtually removed) **A***Z.aprilia* sp. nov. holotype (CASENT0764763) **B***Z.kouakoui* sp. nov. paratype (CASENT0764653) **C***Z.lolae* sp. nov. holotype (KGCOL02270) **D***Z.lumumbai* sp. nov. holotype (MRACFOR0010007) **E***Z.ndouri* sp. nov. holotype (KGCOL01883) **F***Z.obamai* Hita Garcia, 2017 holotype (CASENT0764125) **G***Z.sarowiwai* Hita Garcia, 2017 paratype (CASENT0764650) **H***Z.wilsoni* Hita Garcia, 2017 holotype (MCZ-ENT00512764).

**Figure 12.**
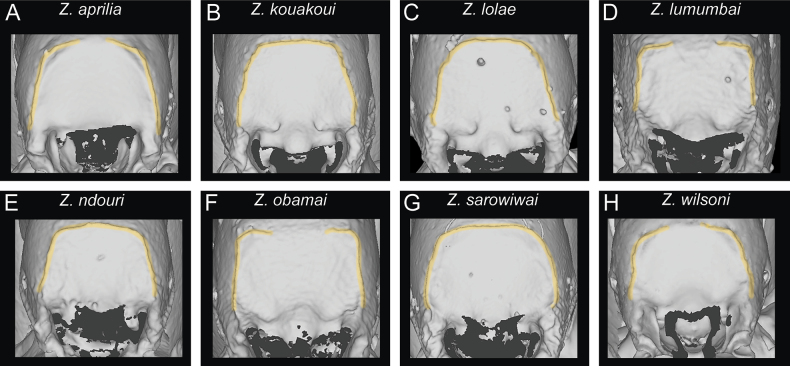
Diagnostic plate showing still images from surface volume renderings of the mesosoma in posterior view focusing on propodeal declivity (outline of declivity in semi-transparent yellow) **A***Z.aprilia* sp. nov. holotype (CASENT0764763) **B***Z.kouakoui* sp. nov. paratype (CASENT0764653) **C***Z.lolae* sp. nov. paratype (CASENT0764651) **D***Z.lumumbai* sp. nov. holotype (MRACFOR0010007) **E***Z.ndouri* sp. nov. holotype (KGCOL01883) **F***Z.obamai* Hita Garcia, 2017 holotype (CASENT0764125) **G***Z.sarowiwai* Hita Garcia, 2017 paratype (CASENT0764650) **H***Z.wilsoni* Hita Garcia, 2017 holotype (MCZ-ENT00512764).

**Figure 13.**
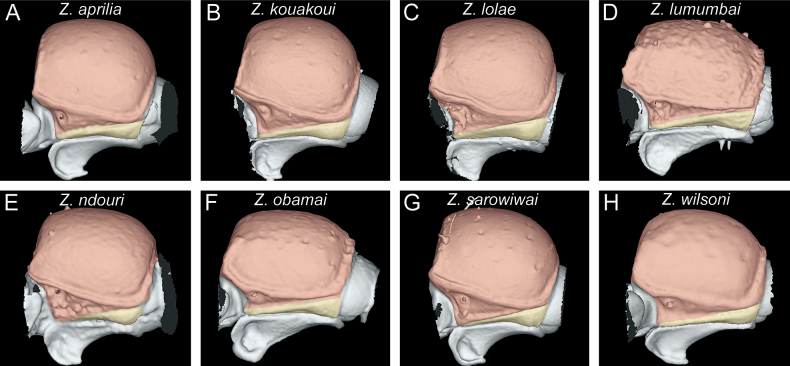
Diagnostic plate showing still images from surface volume renderings of the tergum of AS II (petiole) in profile view (the remainder of the body virtually removed) (tergum in semi-transparent red, laterotergite in semi-transparent yellow) **A***Z.aprilia* sp. nov. holotype (CASENT0764763) **B***Z.kouakoui* sp. nov. paratype (CASENT0764653) **C***Z.lolae* sp. nov. holotype (KGCOL02270) **D***Z.lumumbai* sp. nov. holotype (MRACFOR0010007) **E***Z.ndouri* sp. nov. holotype (KGCOL01883) **F***Z.obamai* Hita Garcia, 2017 holotype (CASENT0764125) **G***Z.sarowiwai* Hita Garcia, 2017 paratype (CASENT0764650) **H***Z.wilsoni* Hita Garcia, 2017 holotype (MCZ-ENT00512764).

**Figure 14.**
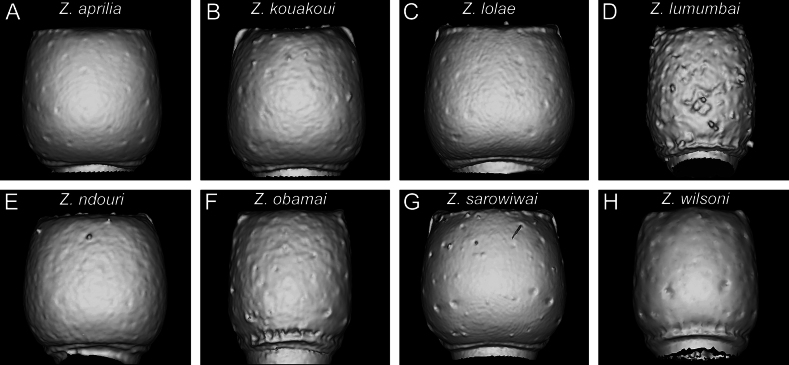
Diagnostic plate showing still images from surface volume renderings of the tergum of AS II (petiole) in dorsal view (the remainder of the body virtually removed) **A***Z.aprilia* sp. nov. holotype (CASENT0764763) **B***Z.kouakoui* sp. nov. holotype (KGCOL00589) **C***Z.lolae* sp. nov. holotype (KGCOL02270) **D***Z.lumumbai* sp. nov. holotype (MRACFOR0010007) **E***Z.ndouri* sp. nov. holotype (KGCOL01883) **F***Z.obamai* Hita Garcia, 2017 holotype (CASENT0764125) **G***Z.sarowiwai* Hita Garcia, 2017 paratype (CASENT0764650) **H***Z.wilsoni* Hita Garcia, 2017 holotype (MCZ-ENT00512764).

**Figure 15.**
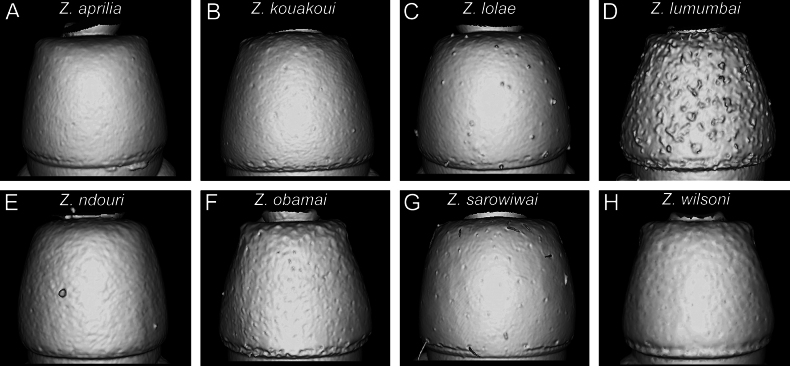
Diagnostic plate showing still images from surface volume renderings of the tergum of AS III in dorsal view (the remainder of the body virtually removed) **A***Z.aprilia* sp. nov. holotype (CASENT0764763) **B***Z.kouakoui* sp. nov. paratype (CASENT0764653) **C***Z.lolae* sp. nov. paratype (CASENT0764651) **D***Z.lumumbai* sp. nov. holotype (MRACFOR0010007) **E***Z.ndouri* sp. nov. holotype (KGCOL01883) **F***Z.obamai* Hita Garcia, 2017 holotype (CASENT0764125) **G***Z.sarowiwai* Hita Garcia, 2017 paratype (CASENT0764650) **H***Z.wilsoni* Hita Garcia, 2017 holotype (MCZ-ENT00512764).

**Figure 16.**
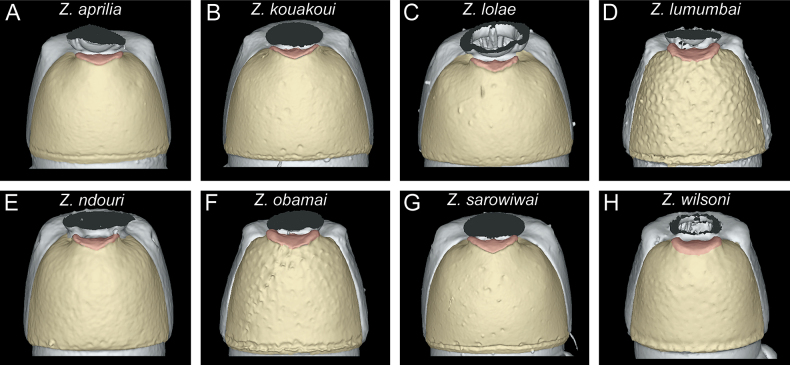
Diagnostic plate showing still images from surface volume renderings of the sternum of AS III in ventral view (the remainder of the body virtually removed) (Bum in semi-transparent yellow, prora in semi-transparent red) **A***Z.aprilia* sp. nov. holotype (CASENT0764763) **B***Z.kouakoui* sp. nov. paratype (CASENT0764653) **C***Z.lolae* sp. nov. paratype (CASENT0764651) **D***Z.lumumbai* sp. nov. holotype (MRACFOR0010007) **E***Z.ndouri* sp. nov. holotype (KGCOL01883) **F***Z.obamai* Hita Garcia, 2017 holotype (CASENT0764125) **G***Z.sarowiwai* Hita Garcia, 2017 paratype (CASENT0764650) **H***Z.wilsoni* Hita Garcia, 2017 holotype (MCZ-ENT00512764).

**Figure 17.**
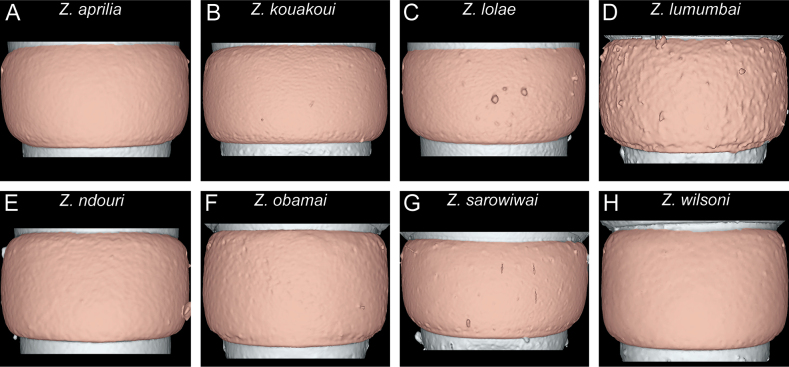
Diagnostic plate showing still images from surface volume renderings of the tergum of AS VI in dorsal view (the remainder of the body virtually removed) (outline of post-sclerite tergum in semi-transparent red) **A***Z.aprilia* sp. nov. holotype (CASENT0764763) **B***Z.kouakoui* sp. nov. paratype (CASENT0764653) **C***Zasphinctuslolae* sp. nov. paratype (CASENT0764651) **D***Z.lumumbai* sp. nov. holotype (MRACFOR0010007) **E***Z.ndouri* sp. nov. holotype (KGCOL01883) **F***Z.obamai* Hita Garcia, 2017 holotype (CASENT0764125) **G***Z.sarowiwai* Hita Garcia, 2017 paratype (CASENT0764650) **H***Z.wilsoni* Hita Garcia, 2017 holotype (MCZ-ENT00512764).

**Figure 18.**
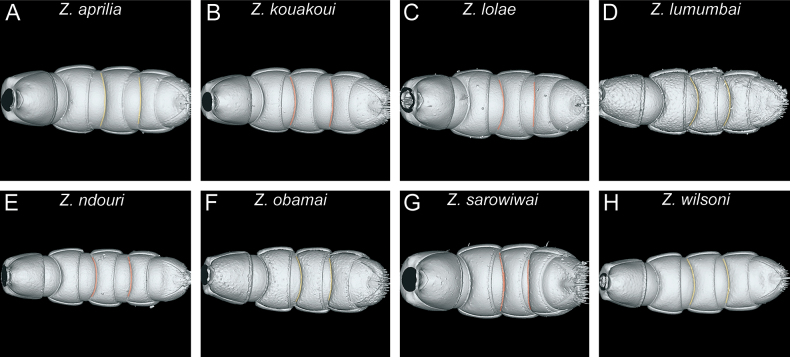
Diagnostic plate showing still images from surface volume renderings of the sternites of AS III-VII in ventral view (the remainder of the body virtually removed) (girdling constrictions with cross-ribbed sculpture are in semi-transparent red; unsculptured girdling constrictions are in semi-transparent yellow) **A***Z.aprilia* sp. nov. holotype (CASENT0764763) **B***Z.kouakoui* sp. nov. paratype (CASENT0764653) **C***Z.lolae* sp. nov. paratype (CASENT0764651) **D***Z.lumumbai* sp. nov. holotype (MRACFOR0010007) **E***Z.ndouri* sp. nov. holotype (KGCOL01883) **F***Z.obamai* Hita Garcia, 2017 holotype (CASENT0764125) **G***Z.sarowiwai* Hita Garcia, 2017 paratype (CASENT0764650) **H***Z.wilsoni* Hita Garcia, 2017 holotype (MCZ-ENT00512764).

In the following, we present compound diagnostic image plates displaying the morphological diversity observed during this study (Figs 4–18). Each body part was virtually sectioned, scaled, and dissected to allow a better examination in 3D. These diagnostic plates are intended to illustrate and aid the new identification key provided below. However, we believe that they also function as a general outline for the morphological characters of taxonomic importance and can be used by future taxonomists, parataxonomists, or ecologists to compare whole specimens or body parts in order to quickly gain a better understanding of the species studied. The full character matrix containing all morphological character states of diagnostic value is available as Suppl. material 1.

#### ﻿Head morphology

The head shape of all species is generally quite similar in being conspicuously much longer than wide, with a CI ranging from 78–86 (Fig. 4). However, there is some observable variation between species and groups. Measuring the head lengths of the *Z.sarowiwai* group species requires including either the clypeal tooth or its neighbouring basal projections of the parafrontal ridges. In contrast, the three species of the *Z.obamai* group do not have any clypeal teeth nor any conspicuous projections nearby. As a consequence, the values for head lengths of the *Z.obamai* group species are a bit shorter than their general head appearance, whereas the head lengths of the *Z.sarowiwai* group species are a bit longer. This means that, despite that the values of CI 78–80 versus CI 80–86 appear to be close and almost overlapping, the head shapes are very clearly different (Fig. 4D, F, H versus Fig. 4A, B, C, E, G). The head shape in lateral view is also overall quite similar but we observed some distinct variation between groups, in particular it appears that some species have a particularly swollen underside (Fig. 5). While we did not undertake any internal anatomical analysis, it appears that the ventral enlargement could be due to additional muscle mass in these species.

The anterior portion of the head bears several morphological structures of importance. Perhaps the most prominent when looking at a *Zasphinctus* head in full-face view is the vertical torular-posttorular complex (Keller 2011), which is typical for the genus in all species (Borowiec 2016). However, there are some pronounced differences in shape and size among species and groups. These differences are noticeable in dorsal (Fig. 4) and lateral view (Fig. 5). Another characteristic of dorylines is the presence of parafrontal ridges (Borowiec 2016), which are very well developed in all species of Afrotropical *Zasphinctus*. The shape seems to be somewhat species-specific but problematic as a diagnostic character (see below for further explanation). Despite the clypeus appearing similar among species, some species possess a conspicuous median tooth, or at least a denticle that is about the same size of the projections of the parafrontal ridges (Fig. 4A, C, E, G).

The posterior part of the head, usually overlooked in most dorylines, shows several interesting morphological structures of diagnostic significance. In particular the development of the vertexal margin (or posterodorsal margin of the head), the vertex itself, the occipital margin, and the occiput are of great importance (Figs 6, 7). In some species there is a very conspicuous and clearly demarcated vertexal margin that separates the frons (or dorsum of the head) from the vertex (posterior face of the head). This margin is clearly visible in posterodorsal and posterior view (Figs 6D, F, H, 7D, F, H). By contrast, the vertexal margin is either fully absent or only very weakly developed in other species (Figs 6A, B, C, E, G, 7A, B, C, E, G). The species with such strong vertexal margin also have the sides of the vertex clearly delimited and the whole vertex appears to be fused to the occiput (Fig. 7D, F, H). Furthermore, the shape and degree of development of the occipital and postoccipital margins shows diagnostic variation between species and groups (Fig. 8). However, despite using the anterior and posterior margins of the occiput, we omit the occiput itself from our diagnostic system. The reason is that it is somewhat difficult to dissect virtually, thus it will be almost impossible to be used by any regular taxonomists or ecologists that do not want to physically destroy their specimens.

As already mentioned above, we observed some conspicuous variability in some morphological structures of the head, not just between species but also within species. Perhaps the most variable is the shape of the parafrontal ridges (Fig. 9). Overall, it seems to be a rather irregular structure that even differs within the same specimen between the left and the right side (Fig. 9A) or among specimens of the same species in its general outline (Fig. 9A, B). But also, many structural details and the development of the anterior projections vary, especially the latter is of interest since it appears to be variable within the same species (Fig. 9A versus Fig. 9B; Fig. 9C versus Fig. 9D). As a consequence, we have omitted characters of the parafrontal ridges from our diagnostic system.

#### ﻿Mesosoma morphology

Overall, the mesosoma of all species is comparatively similar in most characters, except for proportions and few structural details. One important character of the genus is the presence of a pleural endophragmal pit concavity (Borowiec 2016), which was already recognised as having some diagnostic value in the previous revision (Hita Garcia et al. 2017a) (Fig. 10). Also in profile view, there is some noticeable variation in proportions, with some species having a bulkier and higher mesosoma compared to the more elongated one of the other species (Fig. 10). The same differences in proportions are also visible in dorsal view (Fig. 11).

One general characteristic of the genus is the presence of a distinct dorsal margin of the propodeal declivity that is also rectangular in posterior view (Borowiec 2016). This posterodorsal margin of the mesosoma shows some surprising variation since it is medially interrupted or much less strongly developed in several species (Figs 11A, D, F, H, 12A, D, F, H) compared to the others with an intact margin (Figs 11B, C, E, G, 12B, C, E, G).

#### ﻿Metasoma morphology

The tergum and laterotergite of AS II (petiole) are also generally quite similar among the studied species, except for their proportions, appearing lower and elongated in some species (Fig. 13D, F, H) and bulkier in others (Fig. 13A, B, C, E, G). The same phenomenon is also visible in dorsal view (Fig. 14). An important character for all studied species is the subpetiolar process of the petiole (AS II), which is clearly visible in profile (Fig. 13). We observed some variation in the development of the anterior and ventral margins and the presence/absence of a differentiated fenestra. While there are some visible differences among species in ventral view (Hita Garcia et al. 2017a), we do not use the subpetiolar process in that particular perspective since it is often covered by legs, glue, or paper, thus challenging to examine in physical specimens and difficult for virtual dissections. Instead, we think it is useful to observe in profile view and use that perspective.

All species examined possess the typical *Zasphinctus* metasoma with conspicuous girdling constrictions between abdominal segments III, IV, V, and VI. The shape is overall relatively similar with differences only found in characters such as the proportions of some tergites/sternites, grooves at the posterior end of the sternites, the shape and development of the prora, and microsculpture on the constrictions. The metasomal segment of highest diagnostic value is certainly AS III. The tergum of AS III in dorsal view shows some distinct variation among groups by being more trapezoidal in some species (Fig. 15D, F, H) versus more rounded rectangular in others (Fig. 15A, B, C, E, G). The sternum of AS III provides several good diagnostic characters (Fig. 16). The shape of the sternum itself is variable among species and groups, as is the development of the prora, both well visible in ventral view (Fig. 16). In addition, another character is the groove at the posterior end of the sternite, which is highly variable ranging from a thick, deep, sharply, and irregularly outlined transverse groove (Fig. 16D, F), through a thinner, deep, sharply, and relatively regularly outlined transverse groove (Fig. 16A, G), to a weak or absent transverse groove (Fig. 16B, C, E, H).

As in Hita Garcia et al. (2017a), we also measured length and width of AS III to AS VI in order to assess differences in proportions among species. Our measurement data and observations showed little variation, except for AS VI (Fig. 17). The tergum of AS VI is clearly much broader than long in some species (Fig. 17A, B, C, G) contrasting with the less broad tergum seen in others (Fig. 17D, F, H), with one species being intermediate (Fig. 17E). Why only the tergum of AS VI shows such variability compared to AS IV, V, and VII is unknown.

An interesting observation is the presence/absence of the fine, cross-ribbed microsculpture within the girdling constrictions, both dorsally and ventrally, even though usually much weaker dorsally. The species found in Senegal, Ivory Coast, Ghana, and Cameroon all display the cross-ribbed sculpture, whereas it is absent in the species from eastern Congo, Uganda, Kenya, and Mozambique (Fig. 18). Since there are no character states supporting such a geographical division and it seems to be not correlated with the membership to any of the two species groups, we consider this more of a sporadic phenomenon.

### ﻿Afrotropical species groups

The currently known species within the Afrotropical region can be roughly split into two species groups/complexes on the basis of qualitative and quantitative morphology: the *Z.obamai* group and the *Z.sarowiwai* group. We observed an astonishing number of robust differences (we list 18 clear-cut morphological characters in Suppl. material 1) between the members of these groups suggesting that they might very likely represent “genuine” monophyletic groups. However, there could be other explanations, thus without a molecular phylogenetic analysis these two groups are proposed as convenience groups for now. Furthermore, due to us focusing entirely on Afrotropical *Zasphinctus*, it remains to be tested if our groups work in other biogeographical regions.

#### ﻿*Zasphinctusobamai* group

**Figure 19.**
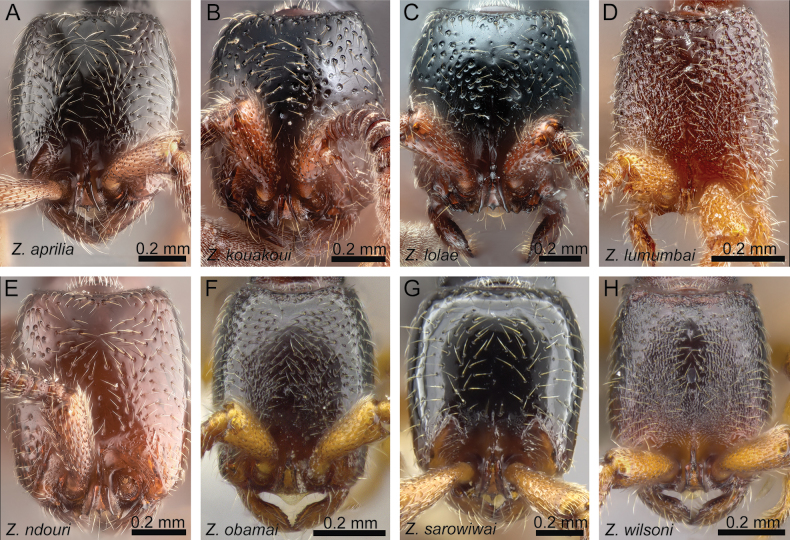
Diagnostic plate showing head in full-face view of all species treated herein (stacked colour images) **A***Z.aprilia* sp. nov. holotype (CASENT0764763) **B***Z.kouakoui* sp. nov. holotype (KGCOL00589) **C***Z.lolae* sp. nov. holotype (KGCOL02270) **D***Z.lumumbai* sp. nov. holotype (MRACFOR0010007) **E***Z.ndouri* sp. nov. holotype (KGCOL01883) **F***Z.obamai* Hita Garcia, 2017 holotype (CASENT0764125) **G***Z.sarowiwai* Hita Garcia, 2017 paratype (CASENT0764650) **H***Z.wilsoni* Hita Garcia, 2017 holotype (MCZ-ENT00512764).

**Figure 20.**
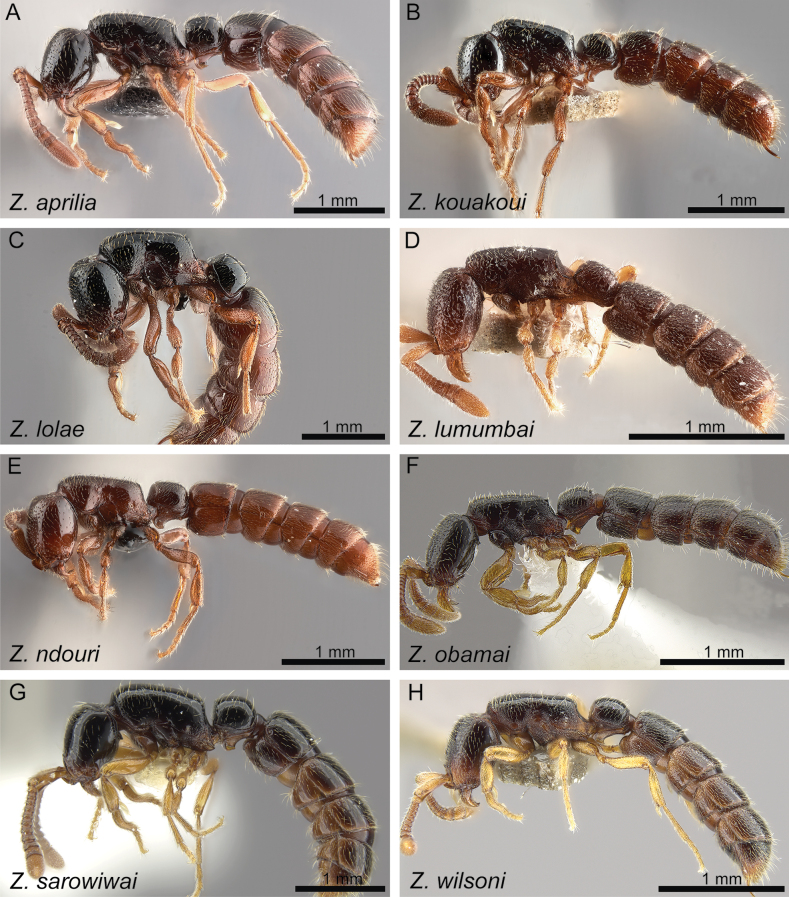
Diagnostic plate showing full body in profile view of all species treated herein (stacked colour images) **A***Z.aprilia* sp. nov. holotype (CASENT0764763) **B***Z.kouakoui* sp. nov. holotype (KGCOL00589) **C***Z.lolae* sp. nov. holotype (KGCOL02270) **D***Z.lumumbai* sp. nov. holotype (MRACFOR0010007) **E***Z.ndouri* sp. nov. holotype (KGCOL01883) **F***Z.obamai* Hita Garcia, 2017 holotype (CASENT0764125) **G***Z.sarowiwai* Hita Garcia, 2017 holotype (CASENT0764654) **H***Z.wilsoni* Hita Garcia, 2017 holotype (MCZ-ENT00512764).

**Diagnosis.** Body size significantly smaller (HL 0.54–0.60; WL 0.73–0.87); head in full-face view appearing thinner (CI 78–80); head in profile appearing conspicuously thinner, its underside only slightly curved; clypeal area always without conspicuous median tooth; torular-posttorular complex in dorsal view with sides more or less parallel; torular-posttorular complex in profile strongly arched anteriorly towards highest dorsal point and posterodorsally lobate; vertexal margin in posterodorsal view strongly developed delimiting posterior face of head; anterior outline of occipital margin in ventral view moderately or weakly and irregularly defined and with anterolateral projections angulate (in *Z.obamai* rounded); vertex with clear margin laterally and not appearing fused to the occiput; anterior outline of postoccipital margin in ventral view moderately or weakly and irregularly defined and with anterolateral projections angulate; mesosoma in profile relatively lower and elongate (LMI 34–37); pleural endophragmal pit strongly developed and deep; petiolar tergum in profile relatively lower (LPI 112-123); mesosoma in dorsal view appearing thinner and elongate (DMI 38–40; DMI2 49–53); petiolar tergum in profile relatively lower, ~ 0.8–0.9 × higher than long (LPI 112–123); petiolar tergum in dorsal view thinner, ~ 0.8–0.9 × broader than long (DPI 82–93); abdominal tergum III in dorsal view strongly trapezoidal with anterior margin more angulate; abdominal sternum III in ventral view rounded trapezoidal, comparatively thinner and higher, sides less rounded; usually with conspicuous surface sculpture somewhere on body (except for piliferous foveae), usually on cephalic dorsum and sides of mesosoma (Figs 19D, F, H, 20D, F, H).

The three species of the *Z.obamai* group appear to be rarer and are only known from Mozambique (*Z.wilsoni*), Kenya (*Z.obamai*) and D.R. of Congo (*Z.lumumbai*), with the latter two species only being found in their respective type localities.

#### ﻿*Zasphinctussarowiwai* group

**Diagnosis.** Body size significantly larger (HL 0.73–0.98; WL 0.98–1.40); head in full-face view appearing thicker (CI 80–86); head in profile appearing conspicuously thicker, with a swollen underside; clypeal area usually with conspicuous median tooth (except for *Z.kouakoui*); torular-posttorular complex in dorsal view with sides converging posteriorly; torular-posttorular complex in profile funnel-shaped; vertexal margin either weakly developed or fully absent; outline of occipital margin in posterodorsal view sharp and very regularly defined; vertex not clearly demarcated and not appearing fused with the occiput; anterior outline of postoccipital margin in ventral view sharp and very regularly defined and with anterolateral projections rounded; mesosoma in profile relatively higher and compact (LMI 37–41); pleural endophragmal pit weakly developed, either shallow but visible or inconspicuous; mesosoma in dorsal view appearing thicker and more compact (DMI 41–45; DMI2 53–59); petiolar tergum in profile relatively higher, ~ 1.0–1.2 × higher than long (LPI 82–108); petiolar tergum in dorsal view thicker, ~ 1.0–1.3 × broader than long (DPI 102–131); abdominal tergum III in dorsal view weakly trapezoidal, more rounded rectangular with less angulate anterior margin; abdominal sternum III in ventral view campaniform, comparatively broader and shorter, sides strongly rounded; usually without any noticeable surface sculpture (except for piliferous foveae) (Figs 19A, B, C, E, G, 20A, B, C, E, G).

**Notes.** The *Z.sarowiwai* group appears to be more species-rich and better sampled since the vast majority of available specimens belong to this group. The distribution range is vast and stretches from Senegal in the west to western Uganda.

### ﻿Identification key to Afrotropical *Zasphinctus* species (workers)

The present work renders the key from Hita Garcia et al. (2017a) fully outdated and in need for revision. Below, we provide a newly developed identification key to the species groups/species of Afrotropical *Zasphinctus*.

**Table:** 

1	Body size significantly larger (HL 0.73–0.98; WL 0.98–1.40); in full-face view head appearing broader (CI 80–86) (Fig. 4A, B, C, E, G); vertexal margin weakly developed or absent (Fig. 6A, B, C, E, G); petiolar node 1.0–1.3 × wider than long (DPI 102–131) (Fig. 14A, B, C, E, G); ASIII in dorsal view appearing more rectangular and less trapezoidal (Fig. 15A, B, C, E, G)	**2 [*Z.sarowiwai* group**]
–	Body size significantly smaller (HL 0.54–0.60; WL 0.73–0.87); in full-face view head appearing thinner (CI 78–80) (Fig. 4D, F, H); vertexal margin strongly developed and clearly separating cephalic dorsum from posterior face of head (Fig. 6D, F, H); petiolar node ~ 1.1–1.2 × longer than wide (DPI 82–93) (Fig. 14D, F, H); ASIII in dorsal view appearing more trapezoidal (Fig. 15D, F, H)	**6 [*Z.obamai* group**]
2	Posterodorsal margin of mesosoma separating propodeal dorsum from declivity interrupted centrally (Figs 11A, 12A); in ventral view girdling constrictions between AS IV, V, and VI unsculptured and not cross-ribbed (Fig. 18A). [D.R. Congo, Uganda]	***Z.aprilia***
–	Posterodorsal margin of mesosoma separating propodeal dorsum from declivity running uninterrupted in straight line (Figs 11B, C, E, G, 12B, C, E, G); in ventral view girdling constrictions between AS IV, V, and VI conspicuously cross-ribbed (Fig. 18B, C, E, G)	**3**
3	In full-face view, head appearing bulkier (Fig. 4G); cephalic dorsum with much less and smaller piliferous foveae (Figs 4G, 6G); torular-posttorular complex in profile comparatively much higher and shaped as relatively wider and larger funnel (Fig. 5G). [Cameroon]	***Z.sarowiwai***
–	In full-face view, head appearing less bulky (Fig. 4B, C, E); cephalic dorsum with many more moderately sized to larger piliferous foveae (Figs 4B, C, E, 6B, C, E); torular-posttorular complex in profile comparatively lower and shaped as relatively narrower and smaller funnel (Fig. 5B, C, E)	**4**
4	With head in full-face view median clypeal area without any tooth or only a very small denticle, if a small denticle is present, its size is significantly smaller than the basal projection of the parafrontal ridges (Fig. 4B); lateral arms of hypostomal carina with a mostly rounded outline (Fig. 8B). [Ivory Coast]	***Z.kouakoui***
–	With head in full-face view median clypeal area usually with conspicuous tooth, its size as big as the basal projection of the parafrontal ridges (Fig. 4C, E); lateral arms of hypostomal carina strongly angulate at widest points (Fig. 8C, E)	**5**
5	Significantly much smaller species (HL 0.73–0.77; WL 0.98–1.05); sternite of AS III appearing only moderately broad and short (Fig. 16E); tergite of AS VI appearing higher, ~ 1.7–1.8 × broader than long (DA6I 168–179) (Fig. 17E); body colouration always much lighter, uniformly reddish brown to moderately darker chestnut brown (Fig. 20E). [Senegal]	***Z.ndouri***
–	Significantly much larger species (HL 0.90–0.98; WL 1.29–1.40); sternite of AS III appearing very broad and short (Fig. 16C); tergite of AS VI appearing shorter, ~ 1.9–2.0 × broader than long (DA6I 189–200) (Fig. 17C); head, mesosoma and petiole always much darker in colour, very dark brown to black, AS III–VI ranging from light to very dark brown (Fig. 20C). [Ghana]	***Z.lolae***
6	Larger species (WL 0.87); with head in full-face view, occipital extensions visible posteriorly as little horns and head widest relatively anteriorly (Fig. 4H); hypostoma with lateral arms strongly diverging, moderately thick, and strongly angulate at widest point (Fig. 8H); prora in ventral view very weakly developed with almost absent lateroventral margins (Fig. 16H); transverse groove at posterior end of abdominal segment III in ventral view weak to absent, instead with irregular groves and rugosity (Fig. 16H). [Mozambique]	***Z.wilsoni***
–	Smaller species (WL 0.73–0.81); with head in full-face view, no occipital extensions visible posteriorly as little horns and head widest relatively medially or posteriorly (Fig. 4D, F); hypostoma with lateral arms less diverging, relatively thin, and mostly rounded throughout (Fig. 8D, F); prora in ventral view well-developed with thick, irregularly shaped and rounded lateroventral margins (Fig. 16D, F); transverse groove at posterior end of abdominal segment III in ventral view with thick, deep, sharply and irregularly outlined transverse groove (Fig. 16D, F)	**7**
7	Piliferous foveae on dorsum and sides of head conspicuously smaller (Figs 4F, 5F, 19F); subpetiolar process of petiole (AS II) in profile with extremely thickened anterior and ventral margins and well-developed concavity with differentiated fenestra (Fig. 13F). [Kenya]	***Z.obamai***
–	Piliferous foveae on dorsum and sides of head conspicuously larger (Figs 4D, 5D, 19D); subpetiolar process of petiole (AS II) in profile with thickened anterior and ventral margins and weak concavity without differentiated fenestra (Fig. 13D). [D.R. Congo]	***Z.lumumbai***

### ﻿Species accounts

#### ﻿*Zasphinctusobamai* group

##### ﻿ Zasphinctus lumumbai

Taxon classificationAnimaliaHymenopteraFormicidae

Hita Garcia & Gómez sp. nov.

343DD3A1-284B-592D-9958-B561F475B882

https://zoobank.org/083C169D-42ED-4A66-9D5A-32DAFF358DC1

Figs 3D , 4D , 5D , 6D , 7D , 8D , 10D , 11D , 12D , 13D , 14D , 15D , 16D , 17D , 18D , 19D , 20D , 21D , 22

###### Type material examined.

***Holotype*** • Pinned worker, Democratic Republic of Congo, Equateur, Mabali, Tsuhapa River (Bikoro Terr.), Foret Inondée, Humus, collection code ANTC39356, IX.1959 (*N. Leleup*) (MRAC: MRACFOR0010007). [specimen re-mounted by KGA 2022]

***Cybertype*** • Dataset of the holotype (MRACFOR0010007) consists of the volumetric raw data (in DICOM format), a 3D surface model (in PLY format), still images of multiple body parts from surface volume renderings of 3D models, stacked digital colour images illustrating head in full-face view, profile, and dorsal views of the body. The data is deposited at Zenodo (https://doi.org/10.5281/zenodo.12593275) and can be freely accessed as virtual representation of the physical holotype. In addition to the data at Zenodo, we also provide a freely accessible 3D surface model at Sketchfab (https://skfb.ly/p7M7p).

###### Differential worker diagnosis.

**Figure 21.**
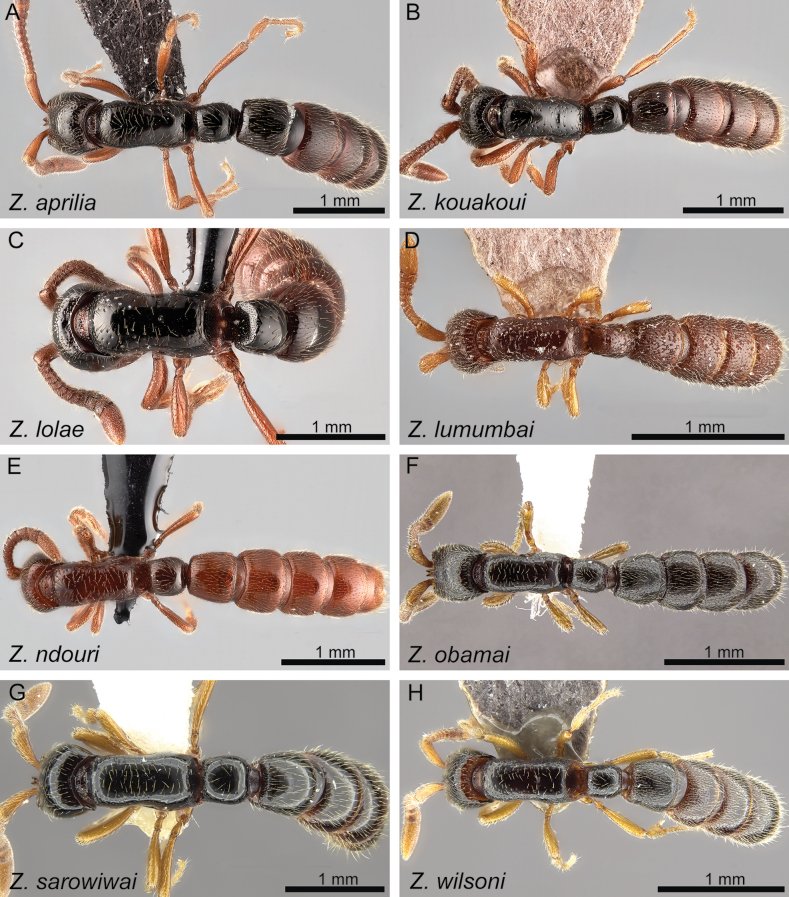
Diagnostic plate showing full body in dorsal view of all species treated herein (stacked colour images) **A***Z.aprilia* sp. nov. holotype (CASENT0764763) **B***Z.kouakoui* sp. nov. holotype (KGCOL00589) **C***Z.lolae* sp. nov. holotype (KGCOL02270) **D***Z.lumumbai* sp. nov. holotype (MRACFOR0010007) **E***Z.ndouri* sp. nov. holotype (KGCOL01883) **F***Z.obamai* Hita Garcia, 2017 holotype (CASENT0764125) **G***Z.sarowiwai* Hita Garcia, 2017 holotype (CASENT0764654) **H***Z.wilsoni* Hita Garcia 2017 holotype (MCZ-ENT00512764).

With characters of the *Z.obamai* group plus the following: body size significantly much smaller (HL 0.54; WL 0.73); lateral arms of hypostomal carina less diverging, relatively thin, and angulate at widest points (Fig. 8D); postgenal sulcus restricted to area adjacent to hypostomal carina and only weakly impressed (Fig. 8D); postoccipital margin in ventral view with anterior outline moderately or weakly and irregularly defined; anterolateral projections angulate (Fig. 8D); pleural endophragmal pit weakly developed and shallow but visible (Fig. 10D); subpetiolar process of petiole (AS II) in profile with thickened anterior and ventral margins and weak concavity without differentiated fenestra (Fig. 13D); posterior end of abdominal segment III in ventral view with thick, deep, sharply and irregularly outlined transverse groove (Fig. 16D); prora in anteroventral view well-developed with thick, irregularly shaped and rounded lateroventral margins (Fig. 16D); surface sculpture on cephalic dorsum and genae mostly smooth and shiny with abundant, relatively deep, and large piliferous foveae, except for reticulate–punctate anteromedian area (Figs 4D, 5D, 19D, 20D); general surface sculpture on mesosoma and metasoma seemingly smooth and shiny with varying degrees of scattered piliferous foveae, hypopygidium reticulate-rugose (Figs 20D, 21D). [general surface sculpture difficult to assess since larger areas are covered in glue and dirt]

###### Measurements and indices.

Morphometric data is based on singleton holotype from the Democratic Republic of Congo and can be seen in Table 2, Suppl. material 3.

**Table 2.** Comparative data of measurements and indices used for the eight species of Afrotropical *Zasphinctus* (raw data is available in Suppl. material 3).

	*Z.aprilia* (N = 6)	*Z.kouakoui* (N = 5)	*Z.lolae* (N = 6)	*Z.lumumbai* (N = 1)	*Z.ndouri* (N = 8)	*Z.obamai* (N = 7)	*Z.sarowiwai* (N = 4)	*Z.wilsoni* (N = 1)
HL	0.84–0.86	0.75–0.80	0.90–0.98	0.54	0.73–0.77	0.55–0.59	0.86–0.89	0.61
HW	0.69–0.73	0.63–0.66	0.77–0.83	0.42	0.59–0.62	0.44–0.47	0.73–0.75	0.49
SL	0.45–0.48	0.40–0.44	0.48–0.54	0.26	0.37–0.38	0.26–0.31	0.48–0.50	0.32
SW	0.17–0.19	0.15–0.18	0.20–0.23	0.12	0.14	0.12–0.14	0.19–0.21	0.12
PH	0.46–0.49	0.40–0.44	0.50–0.54	0.25	0.38–0.39	0.26–0.29	0.48–0.52	0.32
PW	0.50–0.54	0.42–0.46	0.55–0.63	0.29	0.42–0.45	0.28–0.33	0.50–0.53	0.35
DML	0.93–0.98	0.79–0.88	0.95–1.10	0.57	0.79–0.83	0.53–0.65	0.94–0.99	0.66
WL	1.18–1.26	1.03–1.10	1.29–1.40	0.73	0.98–1.05	0.73–0.81	1.20–1.30	0.87
MFL	0.61–0.64	0.55–0.58	0.69–0.75	0.31	0.46–0.51	0.33–0.37	0.62–0.67	0.49
PTL	0.36–0.42	0.33–0.35	0.38–0.44	0.26	0.31–0.34	0.27–0.29	0.44–0.47	0.29
PTH	0.36–0.42	0.36–0.43	0.43–0.48	0.23	0.34–0.35	0.22–0.24	0.42–0.45	0.26
PTW	0.41–0.46	0.36–0.40	0.49–0.53	0.23	0.38–0.40	0.23–0.26	0.45–0.49	0.27
A3L	0.50–0.56	0.45–0.49	0.59–0.68	0.32	0.43–0.46	0.33–0.39	0.53–0.59	0.43
A3W	0.58–0.66	0.50–0.56	0.67–0.75	0.38	0.51–0.55	0.38–0.43	0.62–0.67	0.48
A4L	0.44–0.47	0.39–0.43	0.51–0.57	0.27	0.40–0.43	0.26–0.29	0.50–0.56	0.31
A4W	0.68–0.77	0.66–0.71	0.83–0.90	0.43	0.63–0.67	0.46–0.52	0.77–0.82	0.54
A5L	0.41–0.43	0.35–0.40	0.47–0.51	0.26	0.36–0.38	0.25–0.29	0.44–0.47	0.32
A5W	0.76–0.78	0.68–0.71	0.85–0.92	0.45	0.64–0.67	0.47–0.52	0.78–0.84	0.55
A6L	0.37–0.39	0.33–0.38	0.41–0.44	0.26	0.34–0.37	0.26–0.30	0.37–0.40	0.32
A6W	0.69–0.72	0.66–0.68	0.78–0.84	0.44	0.61–0.64	0.45–0.49	0.73–0.76	0.51
CI	82–85	82–84	83–86	78	80–81	78–80	84–85	82
SI	54–57	53–55	53–55	48	49–51	47–53	56–57	53
SI2	253–282	243–267	239–246	217	264–271	215–242	238–253	267
DMI	42–44	41–43	43–45	40	42–44	38–40	41–42	40
DMI2	53–55	53–54	57–59	51	53–55	48–53	53–55	53
LMI	39–42	38–40	38–39	34	37–39	34–36	40	37
MFI	88–90	86–89	90–92	73	77–83	75–79	86–89	100
LPI	98–102	82–93	86–93	113	92–97	117–123	105–108	112
DPI	102–114	109–114	120–131	88	116–123	82–93	102–109	93
DA3I	113–118	111–116	109–114	119	113–120	108–115	114–117	112
DA4I	154–166	159–173	156–163	159	152–160	170–181	145–158	174
DA5I	178–191	178–200	181–192	173	168–178	174–188	177–179	172
DA6I	178–193	180–204	189–200	169	168–179	163–173	188–197	159

###### Etymology.

The species epithet *lumumbai* is a Latinised noun in the genitive case, named in honour of Mr. Patrice Lumumba, first elected Prime Minister of the Democratic Republic of the Congo.

###### Distribution and biology.

*Zasphinctuslumumbai* is so far only known from one specimen in the MRAC collection, so our knowledge is limited to it being found at the type locality in forest soil.

**Figure 22.**
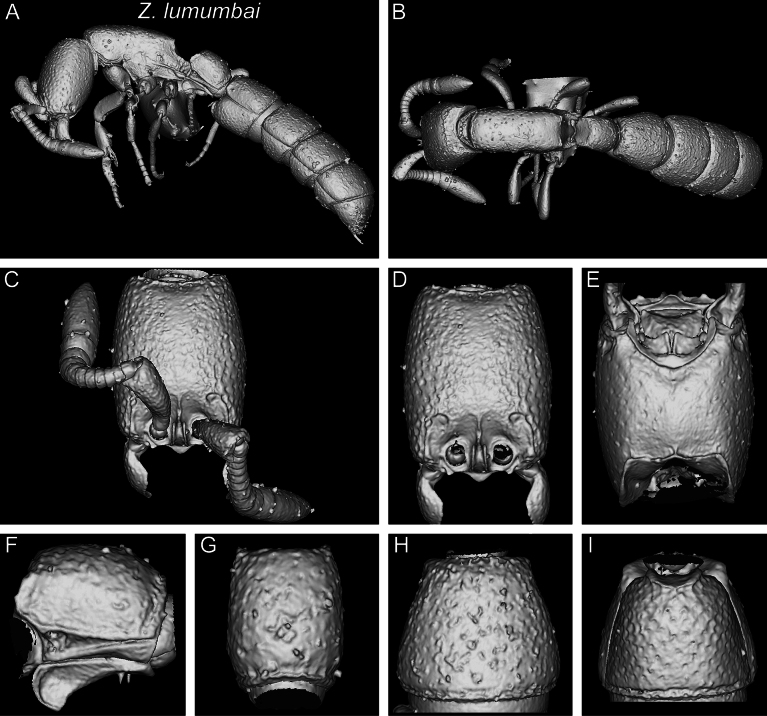
Shaded surface display volume renderings of 3D models of *Z.lumumbai* sp. nov. holotype (MRACFOR0010007) **A** full body in profile **B** full body in dorsal view **C** head in full-face view (with antennae) **D** head in full-face view (without antennae) **E** head in ventral view **F** abdominal segment II (petiole) in profile **G** abdominal segment II (petiole) in dorsal view **H** tergum of AS III in dorsal view **I** sternum of AS III in ventral view.

##### ﻿ Zasphinctus obamai

Taxon classificationAnimaliaHymenopteraFormicidae

Hita Garcia, 2017

122966DB-7B9E-52D1-AA7F-6ADE381208DE

Figs 3F , 4F , 5F , 6F , 7F , 8F , 10F , 11F , 12F , 13F , 14F , 15F , 16F , 17F , 18F , 19F , 20F , 21F , 23

###### Type material examined.

***Holotype*** • Pinned worker, Kenya, Western Province, Kakamega Forest, Buyangu, 0.35222, 34.8647, 1640 m, secondary rainforest, leaf litter, collection code FHG00001, VII.–VIII.2004 (*F. Hita Garcia*) (NMKE: CASENT0764125). ***Paratypes*** • Seven pinned workers: two with same data as holotype (NHMUK: CASENT0764126; MCZC: CASENT0764127) • two from Kenya, Western Province, Kakamega Forest, Isecheno, equatorial rainforest, sifted litter and soil under *Morusmesozygia*, 0.34, 34.85, 1550 m, ANTC8506, 6.XI.2002 (*W. Okeka*) (LACM: CASENT0178218; ZFMK: CASENT0764648) • two from Kenya, Western Province, Kakamega Forest, Kisere Forest Fragment, 0.38505, 34.89378, 1650 m, rainforest, ex leaf litter, Transect 11, collection code FHG00036, 16.VII.2007 (*F. Hita Garcia*) (NMKE: CASENT0764128; NMKE: CASENT0764129) and • one from Kenya, Western Province, Kakamega Forest, Bunyala Forest Fragment, 0.37889, 34.69917, 1448 m, Winkler leaf litter extraction, collection code ANTC39476, VIII.2008 (*G. Fischer*) (ZFMK: CASENT0764647).

***Cybertype*** • Dataset was published in Hita Garcia et al. (2017a) and consists of the volumetric raw data (in DICOM format), 3D PDFs, and 3D rotation videos of scans of head, mesosoma, metasoma, and the full body of the physical holotype (NMKE: CASENT0764125) and/or one paratype (MCZC: CASENT0764127) in addition to montage photos illustrating head in full-face view, profile, and dorsal views of the body of both specimens. The data was deposited at Dryad and can be freely accessed as virtual representation of both types (Hita Garcia et al. 2017c, http://dx.doi.org/10.5061/dryad.4s3v1). In addition to the cybertype data at Dryad, we also provided a freely accessible 3D surface model of the holotype at Sketchfab (https://skfb.ly/6sPvr).

###### Non-type material examined.

• One worker from Kenya, Western Province, Kakamega Forest, Isecheno, equatorial rainforest, sifted litter and soil under *Morusmesozygia*, 0.24, 34.87, 1550 m, collection code ANTC8507, 6.XI.2002 (*W. Okeka*) (LACM: CASENT0178219).

###### Differential worker diagnosis.

With characters of the *Z.obamai* group plus the following: body size significantly much smaller (HL 0.55–0.59; WL 0.73–0.81); lateral arms of hypostomal carina less diverging, relatively thin, and angulate at widest points (Fig. 8F); postgenal sulcus restricted to area adjacent to hypostomal carina and only weakly impressed (Fig. 8F); postoccipital margin in ventral view with anterior outline moderately or weakly and irregularly defined; anterolateral projections rounded (Fig. 8F); pleural endophragmal pit weakly developed and shallow but visible (Fig. 10F); subpetiolar process of petiole (AS II) in profile with extremely thickened anterior and ventral margins and well developed concavity with differentiated fenestra (Fig. 13F); posterior end of abdominal segment III in ventral view with thick, deep, sharply and irregularly outlined transverse groove (Fig. 16F); prora in anteroventral view well-developed with thick, irregularly shaped and rounded lateroventral margins (Fig. 16F); surface sculpture on cephalic dorsum and genae mostly smooth and shiny, with abundant and small piliferous foveae, except for reticulate-punctate anteromedian area (Figs 4F, 5F, 19F, 20F); general surface sculpture on mesosoma and metasoma mostly smooth and shiny with abundant piliferous punctures, except for reticulate-punctate anterior pronotum, mesopleuron, lateral propodeum, most of lateral petiole, and hypopygidium (Figs 20F, 21F)).

###### Measurements and indices.

Morphometric data is based on six workers from Kenya and can be seen in Table 2, Suppl. material 3.

###### Distribution and biology.

*Zasphinctusobamai* is only known from the type locality in western Kenya. As noted in Hita Garcia et al. (2017a), despite a thorough ant inventory (Hita Garcia et al. 2009), it was only collected few times, thus one of the rarest species in that forest system. It was only found in the leaf litter layer of primary or near-primary forest habitats. No new material was collected since its original description. Consequently, *Z.obamai* appears to be endemic to the Kakamega Forest.

**Figure 23.**
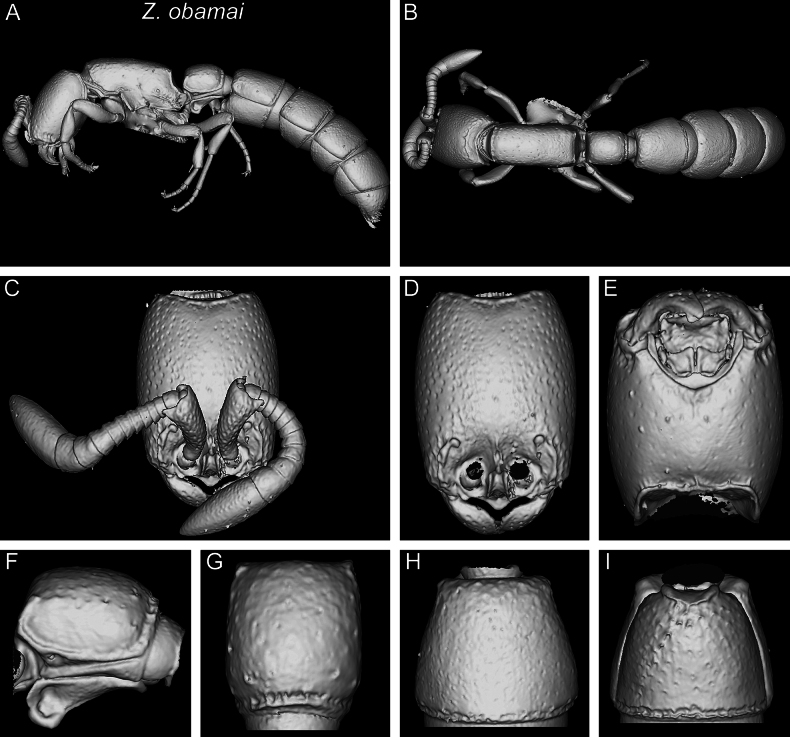
Shaded surface display volume renderings of 3D models of *Z.obamai* Hita Garcia, 2017 holotype (CASENT0764125) **A** full body in profile **B** full body in dorsal view **C** head in full-face view (with antennae) **D** head in full-face view (without antennae) **E** head in ventral view **F** abdominal segment II (petiole) in profile **G** abdominal segment II (petiole) in dorsal view **H** tergum of AS III in dorsal view **I** sternum of AS III in ventral view.

##### ﻿ Zasphinctus wilsoni

Taxon classificationAnimaliaHymenopteraFormicidae

Hita Garcia, 2017

F252AF06-EC07-5B3E-804F-DAF1FB296058

Figs 3H , 4H , 5H , 6H , 7H , 8H , 10H , 11H , 12H , 13H , 14H , 15H , 16H , 17H , 18H , 19H , 20H , 21H , 24

###### Type material examined.

***Holotype*** • Pinned worker, Mozambique, Sofala, Gorongosa National Park, 2 km S Chitengo, -18.99472, 34.35769, 40 m, secondary forest, leaf litter, collection code ANTC37418, 30.V.2012 (*G.D. Alpert*) (MCZC: MCZ-ENT00512764).

***Cybertype*** • Dataset was published in Hita Garcia et al. (2017a) and consists of the volumetric raw data (in DICOM format), as well as 3D PDFs and 3D rotation videos of scans of the head, mesosoma, metasoma, and the full body of the physical holotype (MCZC: MCZ-ENT00512764) in addition to montage photos illustrating head in full-face view, profile, and dorsal views of the body. The data was deposited at Dryad and can be freely accessed as virtual representation of the holotype (Hita Garcia et al. 2017c, http://dx.doi.org/10.5061/dryad.4s3v1). In addition to the cybertype data at Dryad, we also provided a freely accessible 3D surface model of the holotype at Sketchfab (https://skfb.ly/6sPwN).

###### Non-type material examined.

• Five workers from: Mozambique: Cabo Delgado, Parque Nacional Quirimbas, Mareja Reserve, miombo woodland, ex soil, -12.84778, 40.16542, 180 m, collection code BLF38248, 25.II.2016 (*B.L. Fisher; Arthropod Team*) (CASC: CASENT0779283, CASENT0779285) • Zambezia, Mount Mabu, rainforest, ex soil, -16.34888, 36.4081, 375 m, collection code BLF38954, 12.III.2016 (*B.L. Fisher; Arthropod Team*) (CASC: CASENT0779844) • Zambezia, Mount Mabu, rainforest, ex soil, -16.34888, 36.4081, 375 m, collection code BLF39161, 24.III.2016 (*B.L. Fisher; Arthropod Team*) (CASC: CASENT0781222) • Zambezia, Mount Mabu, rainforest, ex soil, -16.34888, 36.4081, 375 m, collection code BLF39209, 25.III.2016 (*B.L. Fisher; Arthropod Team*) (CASC: CASENT0781285).

###### Differential worker diagnosis.

With characters of the *Z.obamai* group plus the following: body size significantly much smaller (HL 0.61; WL 0.87); lateral arms of hypostomal carina strongly diverging anteriorly, relatively thick, and strongly angulate at widest points (Fig. 8H); postgenal sulcus weakly impressed and running halfway to occipital margin (Fig. 8F); postoccipital margin in ventral view with anterior outline moderately or weakly and irregularly defined; anterolateral projections angulate (Fig. 8H); pleural endophragmal pit very weakly developed and inconspicuous (Fig. 10H); subpetiolar process of petiole (AS II) in profile with thickened anterior and ventral margins and weak concavity without differentiated fenestra (Fig. 13H); posterior end of abdominal segment III in ventral view with transverse groove weak to absent, instead with irregular groves and rugosity (Fig. 16H); prora in anteroventral view very weakly developed with almost absent lateroventral margins (Fig. 16H); surface sculpture on genae mostly smooth and shiny, on cephalic dorsum mostly reticulate-rugose (Figs 4H, 5H, 19H, 20H); general surface sculpture on mesosoma and metasoma mostly smooth and shiny with abundant piliferous foveae, mesosoma and petiole laterally mostly reticulate-punctate, hypopygidium reticulate-rugose (Figs 20H, 21H).

###### Measurements and indices.

Morphometric data is based on the singleton holotype from Mozambique and can be seen in Table 2, Suppl. material 3.

###### Distribution and biology.

Fortunately, our knowledge of the distribution of *Z.wilsoni* has increased since its original description. Whereas in Hita Garcia et al. (2017a) it was only known from its type locality, the Gorongosa National Park, now it also known from two additional localities considerably further northeast, namely Quirimbas in Cabo Delgado and Mount Mabu in Zambezia. The species was collected in litter in Gorongosa and in soil in the other two localities. To our surprise it seems that *Z.wilsoni* is relatively flexible in its habitat requirements since it was collected in secondary dry forest, miombo woodland and rainforest.

**Figure 24.**
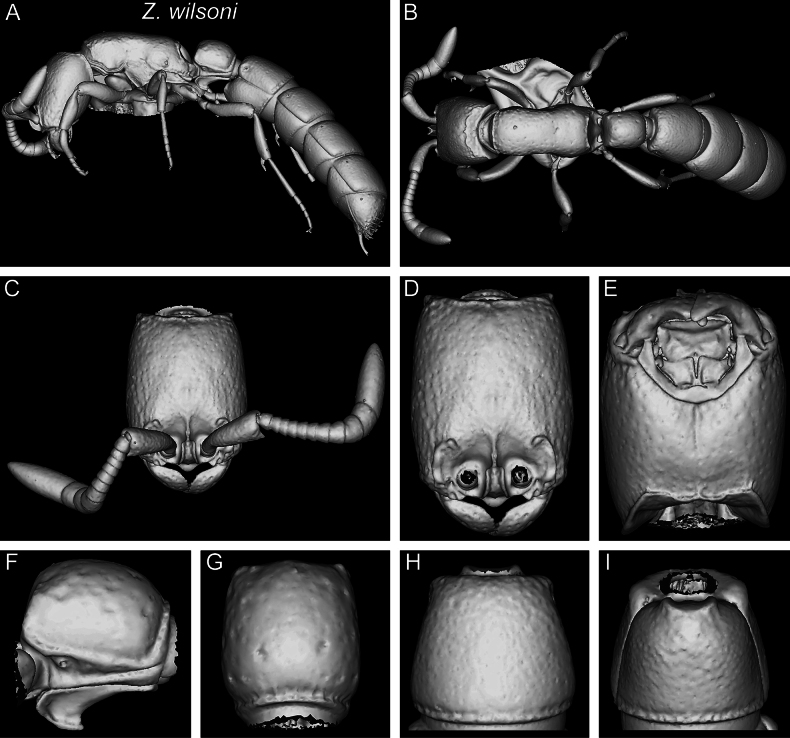
Shaded surface display volume renderings of 3D models of *Z.wilsoni* Hita Garcia, 2017 holotype (MCZ-ENT00512764) **A** full body in profile **B** full body in dorsal view **C** head in full-face view (with antennae) **D** head in full-face view (without antennae) **E** head in ventral view **F** abdominal segment II (petiole) in profile **G** abdominal segment II (petiole) in dorsal view **H** tergum of AS III in dorsal view **I** sternum of AS III in ventral view.

#### ﻿*Zasphinctussarowiwai* group

##### ﻿ Zasphinctus aprilia

Taxon classificationAnimaliaHymenopteraFormicidae

Hita Garcia & Gómez sp. nov.

03D440A0-42DF-5BDF-98A7-03EFE5322230

https://zoobank.org/23504D6E-111E-4D62-B587-1AAFCDBB364B

Figs 2 , 3A , 4A , 5A , 6A , 7A , 8A , 10A , 11A , 12A , 13A , 14A , 15A , 16A , 17A , 18A , 19A , 20A , 21A , 25

###### Type material examined.

***Holotype*** • Pinned worker, Uganda, Kabarole, Kibale National Park, Kanyawara Biological Station, rainforest, ex leaf litter, 0.56437, 30.36059, 1510 m, collection code FHG01047, 6.–16.VIII.2012 (*F. Hita Garcia*) (ZMHB: CASENT0764763). ***Paratypes*** • Three pinned workers from Uganda, Kabarole, Kibale National Park, Kanyawara Biological Station, rainforest opening, field station clearing, hand collected, 0.55838, 30.35992, 1510 m, collection code PGH00079, 6.–16.VIII.2012 (*P.G. Hawkes*) (NHMUK: CASENT0790015; AFRC: CASENT0254676; SAMC: CASENT0254677) and • 2 pinned workers from Uganda, Kabarole, Kibale National Park, Kanyawara Biological Station, moist evergreen forest, ground forager(s), 0.56437, 30.36059, 1520 m, collection codes BLF29378 and BLF29365, 8.VIII.2012 (*B.L. Fisher; F.A. Esteves; Malagasy Arthropod Team*) (CASC: CASENT0352810, CASENT0352813).

***Cybertype*** • Dataset of the holotype (CASENT0764763) consists of the volumetric raw data (in DICOM format), a 3D surface model (in PLY format), still images of multiple body parts from surface volume renderings of 3D models, stacked digital colour images illustrating head in full-face view, profile and dorsal views of the body. The data is deposited at Zenodo (https://doi.org/10.5281/zenodo.12593275) and can be freely accessed as virtual representation of the physical holotype. In addition to the data at Zenodo, we also provide a freely accessible 3D surface model at Sketchfab (https://skfb.ly/p7MpJ).

###### Non-type material examined.

• Twenty workers from: Democratic Republic of Congo: Ituri, Matenda, Label F.98, -1.15653, 27.41793, ca 600 m, 22.IX.1929 (*A. Collart*) (MRAC: MRACFOR000101, MRACFOR000102, MRACFOR000103, MRACFOR000104, MRACFOR000105, MRACFOR000106, MRACFOR000107, MRACFOR000108, MRACFOR000109, MRACFOR000110, MRACFOR000111, MRACFOR000112, MRACFOR000113, MRACFOR000114, MRACFOR000115, MRACFOR000116); Ituri, Madyu (La Moto), 1.77076, 30.29094, ca 1400 m (*L. Burgeon*) (MRAC: MRACFOR001000) • Haut Huelé, Abimva, 3.6452, 29.4306, ca 700 m, 19.–22.VI.1925 (*H. Schouteden*) (MRAC: MRACFOR001144) • Uganda: Kabarole, Kibale National Park, Kanyawara Biological Station, moist evergreen forest, ground forager(s), 0.56437, 30.36059, 1520 m, collection code BLF29365, 8.VIII.2012 (*B.L. Fisher; F.A. Esteves; Malagasy Arthropod Team*) (CASC: CASENT0352811, CASENT0352812).

**Figure 25.**
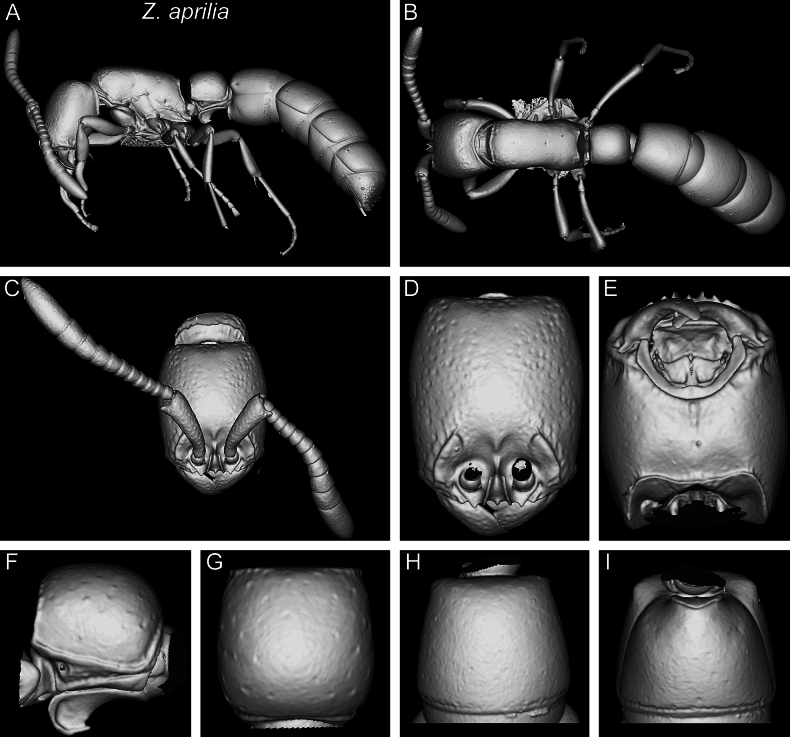
Shaded surface display volume renderings of 3D models of *Z.aprilia* sp. nov. holotype (CASENT0764763) **A** full body in profile **B** full body in dorsal view **C** head in full-face view (with antennae) **D** head in full-face view (without antennae) **E** head in ventral view **F** abdominal segment II (petiole) in profile **G** abdominal segment II (petiole) in dorsal view **H** tergum of AS III in dorsal view **I** sternum of AS III in ventral view.

###### Differential worker diagnosis.

With characters of the *Z.sarowiwai* group plus the following: body size significantly much larger (HL 0.84–0.86; WL 1.18–1.26); torular–posttorular complex in profile comparatively lower and funnel–shaped (Fig. 5A); vertexal margin very weak and dorsum smoothly rounding onto posterior face of head (Figs 6A, 7A); lateral arms of hypostomal carina strongly diverging anteriorly, relatively thick, and strongly angulate at widest points (Fig. 8A); postgenal sulcus weakly impressed and running less than halfway to occipital margin (Fig. 8A); posterodorsal margin of mesosoma interrupted medially (Figs 11A, 12A); subpetiolar process of petiole (AS II) in profile with thickened anterior and ventral margins and well developed concavity with differentiated fenestra (Fig. 13A); petiolar tergum in dorsal view relatively thicker: ~ 1.0–1.1 × broader than long (DPI 102–114) (Fig. 14A); abdominal sternum III in ventral view campaniform, comparatively broad and short, sides strongly rounded (Fig. 16A); posterior end of abdominal segment III in ventral view with thinner, deep, sharply and relatively regularly outlined transverse groove (Fig. 16A); prora in anteroventral view well-developed with sharply and very regularly shaped lateroventral margins (Fig. 16A); abdominal segment VI in dorsal view distinctly shorter: ~ 1.8–1.9 × broader than long (DA6I 178–193) (Fig. 17A); girdling constrictions between abdominal segments IV, V, VI unsculptured (Fig. 18A); surface sculpture on cephalic dorsum and genae completely smooth and very shiny with moderately dense, deep, and moderately sized to large piliferous foveae (Figs 4A, 5A, 19A, 20A); general surface sculpture on mesosoma and metasoma almost completely smooth and very shiny with scattered, piliferous foveae (Figs 20A, 21A).

###### Measurements and indices.

Morphometric data is based on seven workers from Uganda and the Democratic Republic of Congo and can be seen in Table 2, Suppl. material 3.

###### Etymology.

This species is dedicated to Aprilia Selistiowati, the wonderful wife of the first author. The species epithet is to be treated as a noun in apposition.

###### Distribution and biology.

Based on the current data, it seems that *Z.aprilia* has the widest distribution range of all its African congeners since it is known from its type locality in western Uganda, as well as from three additional ones in the eastern parts of the D.R. Congo.

##### ﻿ Zasphinctus kouakoui

Taxon classificationAnimaliaHymenopteraFormicidae

Hita Garcia & Gómez sp. nov.

12BF1247-890B-550E-8431-963B8127DB15

https://zoobank.org/AB56303F-E461-42FB-8303-95CB04ECD0E7

Figs 3B , 4B , 5B , 6B , 7B , 8B , 10B , 11B , 12B , 13B , 14B , 15B , 16B , 17B , 18B , 19B , 20B , 21B , 26

###### Type material examined.

***Holotype*** • Pinned worker, Ivory Coast, Montagnes District, Taï National Park, Site 04, primary forest, hand collected, ex soil, 5.8309, -7.3440, 200 m, collection code KG04079, 10.XI.2019 (*K. Gómez and L. Kouakou*) (RBINS: KGCOL00589). ***Paratypes*** • Four pinned workers with same data as holotype (KGAC: KGCOL00586; MNHNC: KGCOL01321; RBINS: KGCOL02132; NHMUK: KGCOL01884) • 1 pinned worker fromIvory Coast, Tai Forest, 5.83, -7.34, 18.V.77 (*T. Diomande*) (ZMHB: CASENT0764653).

***Cybertype*** • Dataset includes data from the holotype (KGCOL00589) and one paratype (CASENT0764653), and consists of the volumetric raw data (in DICOM format), 3D surface model (in PLY format), still images of multiple body parts from surface volume renderings of 3D models, and stacked digital colour images illustrating head in full-face view, profile, and dorsal views of the body. The data is deposited at Zenodo (https://doi.org/10.5281/zenodo.12593275) and can be freely accessed as virtual representation of the physical holotype and paratype. In addition to the data at Zenodo, we also provide a freely accessible 3D surface model at Sketchfab (https://skfb.ly/p7MpP and https://skfb.ly/p7MpQ).

**Figure 26.**
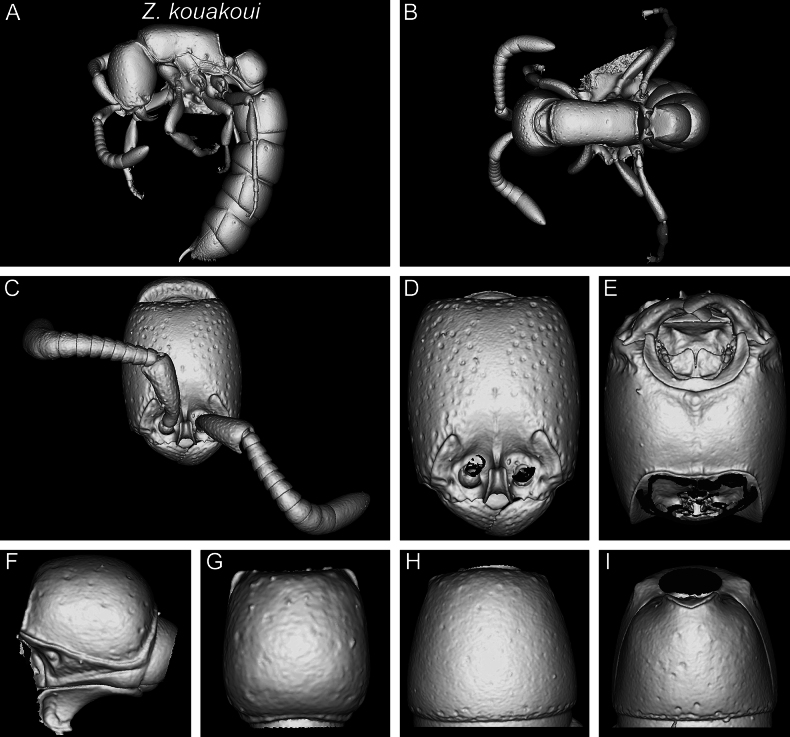
Shaded surface display volume renderings of 3D models of *Z.kouakoui* sp. nov. paratype (CASENT0764653) **A** full body in profile **B** full body in dorsal view **C** head in full-face view (with antennae) **D** head in full-face view (without antennae) **E** head in ventral view **F** abdominal segment II (petiole) in profile **G** abdominal segment II (petiole) in dorsal view **H** tergum of AS III in dorsal view **I** sternum of AS III in ventral view.

###### Differential worker diagnosis.

With characters of the *Z.sarowiwai* group plus the following: body size significantly larger (HL 0.75–0.80; WL 1.03–1.10); torular–posttorular complex in profile comparatively lower and funnel–shaped (Fig. 5B); vertexal margin very weak and dorsum smoothly rounding onto posterior face of head (Figs 6B, 7B); lateral arms of hypostomal carina strongly diverging anteriorly, relatively thick, and outline mostly rounded (Fig. 8B); postgenal sulcus deeply and conspicuously impressed but only running halfway to occipital margin (Fig. 8B); posterodorsal margin of mesosoma continuous across its entire length (Figs 11B, 12B); subpetiolar process of petiole (AS II) in profile with thickened anterior and ventral margins and well developed concavity with differentiated fenestra (Fig. 13B); petiolar tergum in dorsal view relatively thicker: ~ 1.1 × broader than long (DPI 109–114) (Fig. 14B); abdominal sternum III in ventral view campaniform, comparatively broad and short, sides strongly rounded (Fig. 16B); posterior end of abdominal segment III in ventral view with transverse groove weak to absent, instead with irregular groves and rugosity (Fig. 16B); prora in anteroventral view well–developed with sharply and very regularly shaped lateroventral margins (Fig. 16B); abdominal segment VI in dorsal view distinctly shorter: ~ 1.8–2 × broader than long (DA6I 180–204) (Fig. 17B); girdling constrictions between abdominal segments IV, V, VI cross-ribbed (Fig. 18B); surface sculpture on cephalic dorsum and genae completely smooth and very shiny with moderately dense, deep, and moderately sized to large piliferous foveae (Figs 4B, 5B, 19B, 20B); general surface sculpture on mesosoma and metasoma almost completely smooth and very shiny with scattered, piliferous foveae (Figs 20B, 21B).

###### Measurements and indices.

Morphometric data is based on five workers from Ivory Coast and can be seen in Table 2, Suppl. material 3.

###### Etymology.

The species name *kouakoui* is a Latinised noun in the genitive case, dedicated to our good friend and Ivorian myrmecologist Dr. Lombart Kouakou. May this serve as a recognition of his present and future endeavours in Afrotropical myrmecology.

###### Distribution and biology.

Presently, *Z.kouakoui* is only known from two collection events from the type locality, the Tai National Park in Ivory Coast, which is the last remaining major intact block of primary forest in West Africa. It was declared a UNESCO World Heritage Site in 1982 due to exceptional richness in fauna and ﬂora. Indeed, based on several criteria including species diversity, endemism, presence of rare species, and/or endangered and critical habitats, the Tai National Park is considered a priority for the conservation of mammals, birds, amphibians, and invertebrates in West Africa (Reizebos et al. 1994).

##### ﻿ Zasphinctus lolae

Taxon classificationAnimaliaHymenopteraFormicidae

Hita Garcia & Gómez sp. nov.

F959CFF0-5EEB-5C02-9DB8-BF256EB7AEFA

https://zoobank.org/B9A5C45C-DE85-42BC-891E-785BC5C65EB3

Figs 3C , 4C , 5C , 6C , 7C , 8C , 9C , 9D , 10C , 11C , 12C , 13C , 14C , 15C , 16C , 17C , 18C , 19C , 20C , 21C , 27

###### Type material examined.

***Holotype*** • Pinned worker, Ghana, Bobiri Forest Reserve, primary unlogged forest, hand collected, ex soil, 6.69048, -1.33828, ca 260 m, collection code KG03946, 10.I.2019 (*K. Gómez*) (RBINS: KGCOL02270). ***Paratypes*** • Three pinned workers with same data as holotype (KGAC: KGCOL02269; MNHNC: KGCOL00163; RBINS: CASENT0881885) • two pinned workers from Ghana, Wiawso, ant ecology sample, 6.915525, -2.03919, ca 300 m, collection code ANTC39479, 25.IV.1969 (*D. Leston*) (NHMUK: CASENT0764652; ZMHB: CASENT0764651).

***Cybertype*** • Dataset includes data from the holotype (KGCOL02270) and one paratype (CASENT0764651), and consists of the volumetric raw data (in DICOM format), 3D surface model (in PLY format), still images of multiple body parts from surface volume renderings of 3D models, and stacked digital colour images illustrating head in full-face view, profile and dorsal views of the body. The data is deposited at Zenodo (https://doi.org/10.5281/zenodo.12593275) and can be freely accessed as virtual representation of the physical holotype and paratype. In addition to the data at Zenodo, we also provide two freely accessible 3D surface models at Sketchfab (https://skfb.ly/p7MpV and https://skfb.ly/p7MpW).

**Figure 27.**
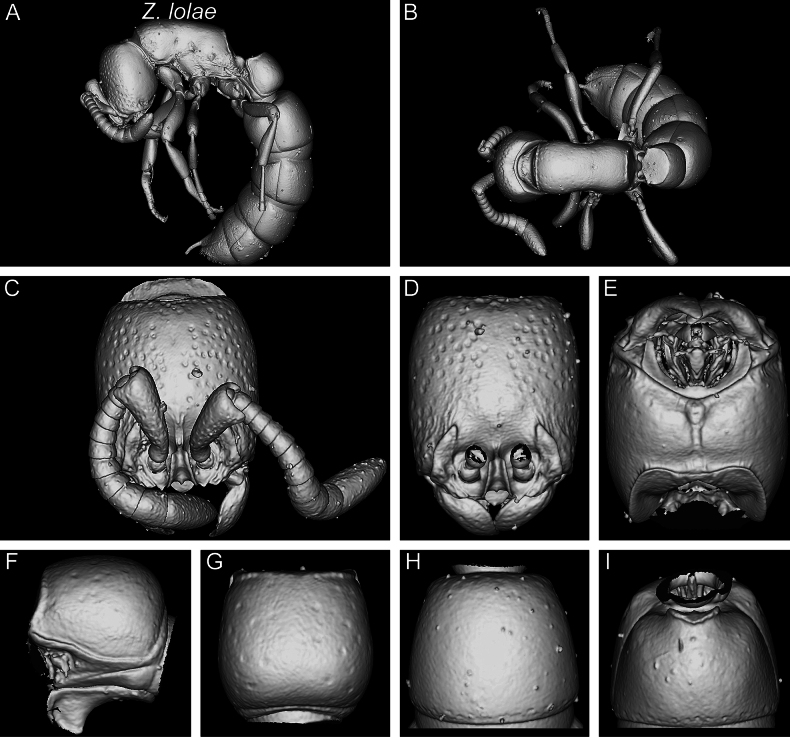
Shaded surface display volume renderings of 3D models of *Z.lolae* sp. nov. holotype (KGCOL02270) **A** full body in profile **B** full body in dorsal view **C** head in full-face view (with antennae) **D** head in full-face view (without antennae) **E** head in ventral view **F** abdominal segment II (petiole) in profile **G** abdominal segment II (petiole) in dorsal view **H** tergum of AS III in dorsal view **I** sternum of AS III in ventral view.

###### Differential worker diagnosis.

With characters of the *Z.sarowiwai* group plus the following: body size significantly much larger (HL 0.90–0.98; WL 1.29–1.40); torular-posttorular complex in profile comparatively lower and funnel-shaped (Fig. 5C); vertexal margin and posterior face of head weakly developed (Figs 6C, 7C); lateral arms of hypostomal carina strongly diverging anteriorly, relatively thick, and strongly angulate at widest points (Fig. 8C); postgenal sulcus deeply and conspicuously impressed and running almost to occipital margin (Fig. 8C); posterodorsal margin of mesosoma continuous across its entire length (Figs 11C, 12C); subpetiolar process of petiole (AS II) in profile with thickened anterior and ventral margins and well developed concavity with differentiated fenestra (Fig. 13C); petiolar tergum in dorsal view relatively thicker: ~ 1.2–1.3 × broader than long (DPI 120–131) (Fig. 14C); abdominal sternum III in ventral view campaniform, very broad and short, sides strongly rounded (Fig. 16C); posterior end of abdominal segment III in ventral view with transverse groove weak to absent, instead with irregular groves and rugosity (Fig. 16C); prora in anteroventral view well-developed with sharply and very regularly shaped lateroventral margins (Fig. 16C); abdominal segment VI in dorsal view distinctly shorter: ~ 1.9–2 × broader than long (DA6I 189–200) (Fig. 17C); girdling constrictions between abdominal segments IV, V, VI cross-ribbed (Fig. 18C); surface sculpture on cephalic dorsum and genae completely smooth and very shiny with moderately dense, deep, and moderately sized to large piliferous foveae (Figs 4C, 5C, 19C, 20C); general surface sculpture on mesosoma and metasoma almost completely smooth and very shiny with scattered, piliferous foveae (Figs 20C, 21C).

###### Measurements and indices.

Morphometric data is based on six workers from Ghana and can be seen in Table 2, Suppl. material 3.

###### Etymology.

The species name *lolae* is a Latinised noun in the genitive case, dedicated to the mother of the second author Kiko Gomez. Thanks for everything.

###### Distribution and biology.

Presently, *Z.lolae* is only known from two collection events from Ghana, from Wiawso and Bobiri Forest Reserve, both of which are/were rainforest habitats.

[Note: the 3D model of the mouthparts presented in Hita Garcia et al. (2017a) is not *Z.sarowiwai*, but instead *Z.lolae* (CASENT0764652)]

##### ﻿ Zasphinctus ndouri

Taxon classificationAnimaliaHymenopteraFormicidae

Hita Garcia & Gómez sp. nov.

41DA750F-A9AA-520B-9DB8-4C0E4CB7A500

https://zoobank.org/D714D293-666C-407D-AC7C-91C8C619F1E5

Figs 3E , 4E , 5E , 6E , 7E , 8E , 10E , 11E , 12E , 13E , 14E , 15E , 16E , 17E , 18E , 19E , 20E , 21E , 28

###### Type material examined.

***Holotype*** • Pinned worker, Senegal, Kedougou, Neménick, Niokolo Koba 10 Km W (Niokolo Koba NP), savannah, Winkler, 13.0764, -12.78196, collection code KG05413, 1.-30.IV.2018 (*A. Diallo*) (RBINS: KGCOL01883). ***Paratypes*** • Eight pinned workers with same data as holotype (CASC: KGCOL02264; KGAC: KGCOL02258; MNHNC: KGCOL02259; NHMUK: KGCOL02262; RBINS: KGCOL02260; SAMC: KGCOL02263; ZMHB: KGCOL02261).

***Cybertype*** • Dataset of the holotype (KGCOL01883) consists of the volumetric raw data (in DICOM format), 3D surface model (in PLY format), still images of multiple body parts from surface volume renderings of 3D models, stacked digital colour images illustrating head in full-face view, profile and dorsal views of the body. The data is deposited at Zenodo (https://doi.org/10.5281/zenodo.12593275) and can be freely accessed as virtual representation of the physical holotype. In addition to the data on Zenodo, we also provide a freely accessible 3D surface model at Sketchfab (https://skfb.ly/p7MpY).

###### Differential worker diagnosis.

With characters of the *Z.sarowiwai* group plus the following: body size significantly larger (HL 0.73–0.77; WL 0.98–1.05); torular–posttorular complex in profile comparatively lower and funnel–shaped (Fig. 5E); vertexal margin very weak and dorsum smoothly rounding onto posterior face of head (Figs 6E, 7E); lateral arms of hypostomal carina strongly diverging anteriorly, relatively thick, and strongly angulate at widest points (Fig. 8E); postgenal sulcus weakly impressed and running halfway to occipital margin (Fig. 8E); posterodorsal margin of mesosoma continuous across its entire length (Figs 11E, 12E); subpetiolar process of petiole (AS II) in profile only weakly thickened anterior and ventral margins, weak concavity and no conspicuous fenestra (Fig. 13E); petiolar tergum in dorsal view relatively thicker: ~ 1.2 × broader than long (DPI 116–123) (Fig. 14E); abdominal sternum III in ventral view campaniform, comparatively broad and short, sides strongly rounded (Fig. 16E); posterior end of abdominal segment III in ventral view with transverse groove weak to absent, instead with irregular groves and rugosity (Fig. 16E); prora in anteroventral view well-developed with sharply and very regularly shaped lateroventral margins (Fig. 16E); abdominal segment VI in dorsal view moderately sized: around 1.7–1.8 × broader than long (DA6I 168–179) (Fig. 17E); girdling constrictions between abdominal segments IV, V, VI cross-ribbed (Fig. 18E); surface sculpture on cephalic dorsum and genae completely smooth and very shiny with moderately dense, deep, and moderately sized to large piliferous foveae (Figs 4E, 5E, 19E, 20E); general surface sculpture on mesosoma and metasoma almost completely smooth and very shiny with scattered, piliferous foveae (Figs 20E, 21E).

###### Measurements and indices.

Morphometric data is based on eight workers from Senegal and can be seen in Table 2, Suppl. material 3.

###### Etymology.

The species epithet is a Latinised noun in the genitive case, dedicated to the Senegalese activist, composer, and musician Youssou N’Dour.

###### Distribution and biology.

Currently, *Z.ndouri* is only known from its type locality, the Niokolo Koba National Park in Senegal. Unlike the other species treated herein, *Z.ndouri* was found in a tropical savanna habitat.

**Figure 28.**
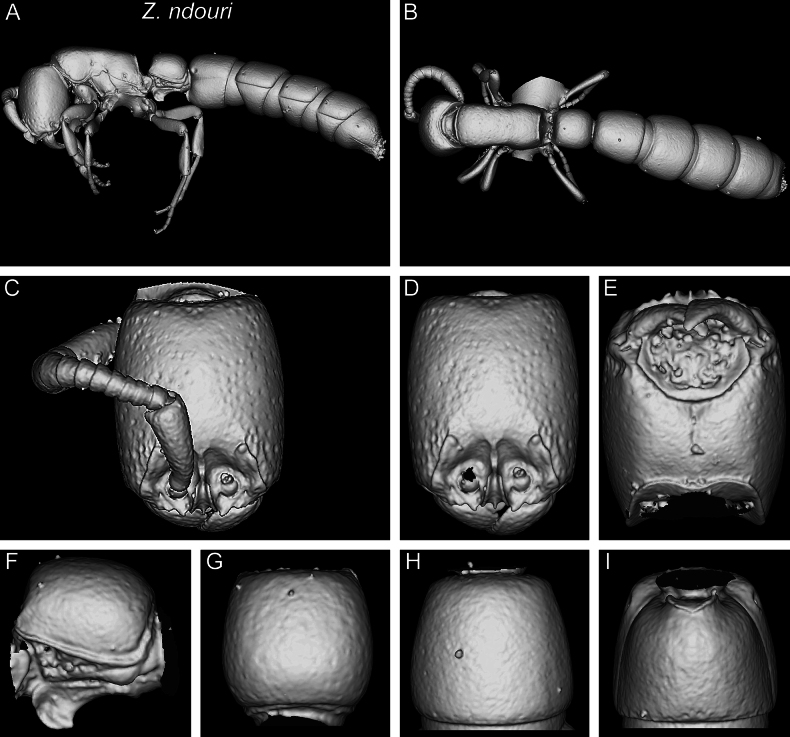
Shaded surface display volume renderings of 3D models of *Z.ndouri* sp. nov. holotype (KGCOL01883) **A** full body in profile **B** full body in dorsal view **C** head in full-face view (with antennae) **D** head in full-face view (without antennae) **E** head in ventral view **F** abdominal segment II (petiole) in profile **G** abdominal segment II (petiole) in dorsal view **H** tergum of AS III in dorsal view **I** sternum of AS III in ventral view.

##### ﻿ Zasphinctus sarowiwai

Taxon classificationAnimaliaHymenopteraFormicidae

Hita Garcia, 2017

C4D4F03D-5530-5C98-AF56-B7D495EA3B31

Figs 3G , 4G , 5G , 6G , 7G , 8G , 9A , 9B , 10G , 11G , 12G , 13G , 14G , 15G , 16G , 17G , 18G , 19G , 20G , 21G , 29

###### Type material examined.

***Holotype*** • Pinned worker, Cameroon, Centre Province, Mbalmayo, 3.4597, 11.4714, ca 600 m, rainforest, collection code ANTC39478, XI.1993 (*N. Stork*) (NHMUK: CASENT0764654). ***Paratypes*** • three pinned workers with same data as holotype (NHMUK: CASENT0764646; CASENT0764649; CASENT0764650).

***Cybertype*** • Dataset was published in Hita Garcia et al. (2017a) and consists of the volumetric raw data (in DICOM format), as well as 3D PDFs and 3D rotation videos of scans of the head, mesosoma, metasoma, and the full body of the physical holotype (CASENT0764654) and paratype (CASENT0764650) in addition to montage photos illustrating head in full-face view, and profile and dorsal views of the body. The data was deposited at Dryad and can be freely accessed as virtual representation of the holotype (Hita Garcia et al. 2017c; http://dx.doi.org/10.5061/dryad.4s3v1). In addition to the cybertype data at Dryad, we also provided two freely accessible 3D surface models of the holotype and paratype at Sketchfab (https://skfb.ly/6sQwn and https://skfb.ly/oX9VO).

###### Non-type material examined.

• One worker from: Cameroon, Centre Province, Mbalmayo, 3.4597, 11.4714, ca 600 m, rainforest, collection code ANTC39478, XI.1993 (*N. Stork*) (NHMUK: CASENT0900310).

###### Differential worker diagnosis.

With characters of the *Z.sarowiwai* group plus the following: body size significantly much larger (HL 0.86–0.89; WL 1.20–1.30); torular–posttorular complex in profile comparatively much higher and funnel–shaped, funnel comparatively wider (Fig. 5G); vertexal margin and posterior face of head weakly developed (Figs 6G, 7G); lateral arms of hypostomal carina strongly diverging anteriorly, relatively thick, and outline mostly rounded (Fig. 8G); postgenal sulcus deeply impressed and running halfway to occipital margin (Fig. 8G); posterodorsal margin of mesosoma continuous across its entire length (Figs 11G, 12G); subpetiolar process of petiole (AS II) in profile with thickened anterior and ventral margins and well developed concavity with differentiated fenestra (Fig. 13G); petiolar tergum in dorsal view relatively thicker, ~ 1.0–1.1 × broader than long (DPI 102–109) (Fig. 14G); abdominal sternum III in ventral view campaniform, comparatively broad and short, sides strongly rounded (Fig. 16G); posterior end of abdominal segment III in ventral view with thinner, deep, sharply and relatively regularly outlined transverse groove (Fig. 16G); prora in anteroventral view well-developed with sharply and very regularly shaped lateroventral margins (Fig. 16G); abdominal segment VI in dorsal view distinctly shorter: ~ 1.9–2 × broader than long (DA6I 188–197) (Fig. 17G); girdling constrictions between abdominal segments IV, V, VI cross-ribbed (Fig. 18G); surface sculpture on cephalic dorsum and genae completely smooth and very shiny with widely scattered and small piliferous foveae (Figs 4G, 5G, 19G, 20G); general surface sculpture on mesosoma and metasoma almost completely smooth and very shiny with scattered, piliferous foveae (Figs 20G, 21G).

###### Measurements and indices.

Morphometric data is based on four workers from Cameroon and can be seen in Table 2, Suppl. material 3.

###### Distribution and biology.

Compared to its original description in Hita Garcia et al. (2017a), the distribution range of *Z.sarowiwai* appears to be significantly smaller. While initially thought to occur from Ivory Coast to Uganda, at present it is only known from Cameroon (see Discussion below for further details).

**Figure 29.**
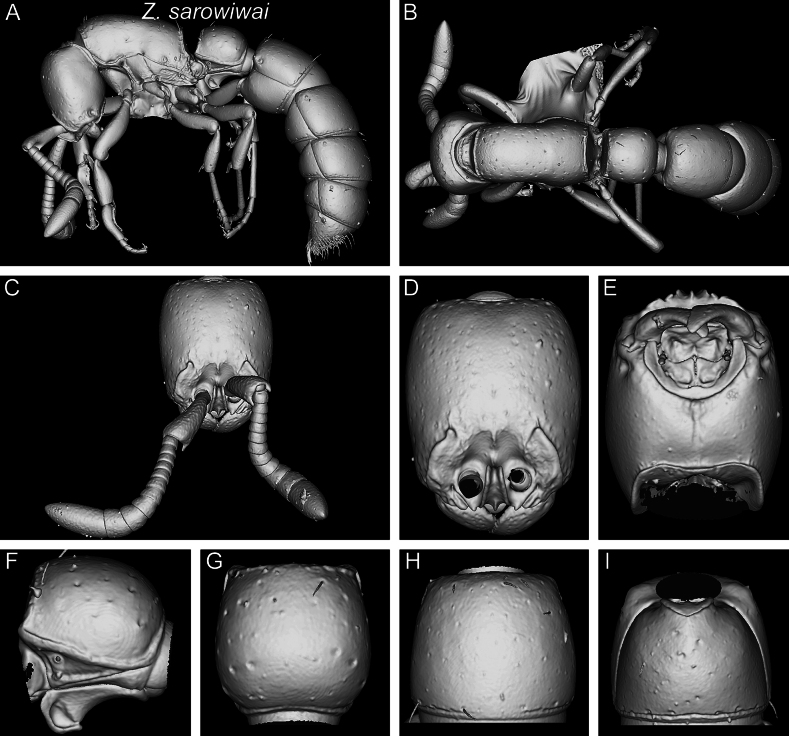
Shaded surface display volume renderings of 3D models of *Z.sarowiwai* Hita Garcia, 2017 holotype (CASENT0764654) **A** full body in profile **B** full body in dorsal view **C** head in full-face view (with antennae) **D** head in full-face view (without antennae) **E** head in ventral view **F** abdominal segment II (petiole) in profile **G** abdominal segment II (petiole) in dorsal view **H** tergum of AS III in dorsal view **I** sternum of AS III in ventral view.

## ﻿Discussion

### ﻿Diversity of Afrotropical *Zasphinctus*

Despite this current work in which we describe five new species, *Zasphinctus* still remains a rarely collected genus. The underlaying material for *Z.kouakoui*, *Z.lolae*, and *Z.ndouri* consists of just one or two collection events, and *Z.lumumbai* is even based on a singleton. The situation for the material of *Z.aprilia* is better since it is from three localities in the northeast of the D.R. Congo and one in western Uganda, but on a larger scale this is still rather limited. This scarcity of material limits our understanding of the genus regarding intraspecific and interspecific variability and geographic distribution.

Prior to this study, *Z.sarowiwai* was considered a single species with a relatively wide distribution, ranging through Equatorial Africa from Ivory Coast in the west to Uganda in the east. Our study with additional new material shows that it indeed consists of four species (“genuine” *Z.sarowiwai* plus *Z.aprilia*, *Z.kouakoui*, and *Z.lolae*), each of them more locally distributed. Thus, one might think that *Zasphinctus* species in general have more restricted geographic distributions than previously thought (Hita Garcia et al. 2017a), but that assessment could also be due to sampling bias. A good example is *Z.wilsoni*, which was only known from one locality in Mozambique, but is now known to occur also much further northeast in the country, thus not particularly endemic anymore. If this is the case for most other species that are currently only known from their type locality remains to be seen with further sampling in the region.

As noted above, the Afrotropical region is a centre for ant diversity (e.g., Guénard et al. 2012), but due to a severe lack of sampling and qualified taxonomists, our understanding of that diversity is fragmentary at best. The recent taxonomic history of *Zasphinctus* is a great example. Prior to Hita Garcia et al. (2017a) the genus was only known from two male-based species. The few known workers in collections were all wrongly identified as *Z.rufiventris*, and overall, there was no worker-based taxonomic system. Hita Garcia et al. (2017a) added three new worker-based species, which are very unlikely to be conspecific with the two previously known male-based forms, thus more than doubling the species count for the genus to five species. Just a few years later, we again double that number to a total of ten.

We believe this development for a comparatively small genus to be a symptom of severely underestimating Afrotropical ant diversity generally. Unlike other regional hotspots, such as the Neotropics or Madagascar, the Afrotropics remain vastly unknown, with vast regions, such as the Congo Basin (3.4 million square kilometres) virtually unsampled. Recent expeditions to few localities in Senegal, Ghana, and Ivory Coast that employed modern collection techniques, such as Winkler leaf litter sampling, have been one main source for most of the new specimens used in this study. As a consequence, we cannot emphasise enough the need for larger scale sampling efforts throughout the region.

Surprisingly the second source of crucial specimens for this study resulted to be the extensive historic material from the D.R. Congo housed in the natural history collections of the Royal Belgian Institute of Natural Sciences in Brussels and the Royal Museum for Central Africa in Tervuren, both in Belgium. Research in these collections unearthed the singleton of *Z.lumumbai* and all of the *Z.aprilia* specimens from the D.R. Congo. Many taxonomic studies are done with material from very few sources of predominantly freshly collected material, and “old” museum collections are often overlooked or excluded. Reasons for this are manifold and involve funding and staff problems at natural history museums, time and funding constraints of taxonomists, and unawareness of the existence of such key material due to lack of public databases or other digitisation sources. Working with historic collections is especially relevant in our case since the northeastern provinces of the D.R. Congo are presently extremely dangerous rendering any field work activities challenging in the best cases. Therefore, we consider researching, funding, and making available the historic collections of natural history museums as imperative to improve our understanding of Afrotropical ant diversity.

### ﻿Virtual recovery of morphology

This study provides a degree of detail and resolution comparable to Hita Garcia et al. (2019), but falls short of the level of resolution of the first *Zasphinctus* revision that included higher resolution micro-CT scans of individual body parts (head, mesosoma, and metasoma) in addition to moderate resolution full-body scans (Hita Garcia et al. 2017a). Due to lower CT scanner availability, we only performed full-body scans for the five new species described herein. In order to avoid problems with comparability, we also only used the full-body scans of the three previously described species. However, as already outlined in Hita Garcia et al. (2019), our approach has some disadvantages. While general morphology is quite accurate in our 3D models, surface sculpture, especially fine microsculpture, is less well recovered in some species. Still, surface sculpture is generally clearly visible in the stacked digital colour images provided. Another disadvantage encountered can be seen in the 3D models of *Z.lumumbai*, which is based on a singleton holotype that is particularly dirty and partly also covered in glue. As a consequence, it was not possible to clean the specimen beforehand and the surface quality of the 3D model is less detailed compared to the other species used in this study. Nonetheless, despite some disadvantages, the achieved resolution of our 3D models is fully acceptable for the comparison of morphological shape data among *Zasphinctus* species.

### ﻿Virtual character evaluation and presentation

One aim of our study was to evaluate the taxonomic delimitation system provided in Hita Garcia et al (2017a), test if its morphological characters still work against the background of many more species and material, and assess if some characters need to be omitted and others included. As noted above, overall, most characters from Hita Garcia et al (2017a) still performed rather well and provided their usefulness in either discriminating between species or species groups. However, we suggest some characters to be discarded, either because they are difficult to describe, visualise, or examine (e.g., antennal scape, occiput in ventral view), or exhibit some form of intraspecific variation (e.g., parafrontal ridges). Alternatively, we recommend the use of some characters previously not used to be of diagnostic importance, but we also discovered some never used before.

Following previous studies (Hita Garcia et al. 2017a, 2019), our choice of characters targets diverse taxonomic audiences. We use many characters that can be easily observed under a microscope with moderate magnification (e.g., shapes of head, mesosoma, and petiole, as well as surface sculpture) for users with restricted microscopy resources or limited taxonomic background. For more taxonomically experienced users with better microscopy resources and deeper knowledge of ant morphology we also offer a plethora of additional, rarely used morphological characters located on the posterior or ventral faces of the head, or the ventral metasoma. The combination of both character sets presented in numerous high resolution diagnostic plates yields a high degree of illustrative power that will greatly enhance taxonomic identifications and future taxonomic revisions, but also serve as comparative data for future systematic studies.

In terms of above-species level characters, we provide almost 20 morphological differences that clearly separate the *Z.obamai* group from the *Z.sarowiwai* group (Suppl. material 1), which is a substantial degree of morphological divergence. Even though currently we do not have any molecular data available, based on such high number of diverging character states we predict that both species groups likely represent monophyletic entities. Considering species-level taxonomy, we used almost 20 morphological characters for species delimitations, thus permitting the generation of diagnoses that are limited to only diagnostically relevant data, but still provide a high level of morphological details.

### ﻿Cybertypes

As in previous studies (e.g., Agavekar et al. 2017; Hita Garcia et al. 2017a, 2017b, 2019; Staab et al. 2018; Sarnat et al. 2019; Sharaf et al. 2019; Gómez et al. 2022), we provide freely available cybertype datasets of all holotypes from all new species described herein (plus additional paratypes for some species), as well as from the previously described species (Hita Garcia et al. 2017c). However, as noted above and in contrast to the previous studies mentioned above, we no longer include 3D PDFs or 3D rotation video files in order to avoid too much data redundancy. The use of 3D videos has been rather prominent in taxonomic studies applying micro-CT data (e.g., Stoev et al. 2013; Akkari et al. 2015, 2018; Fischer et al. 2016; Hita Garcia et al. 2017a, 2019), and we still believe that from a visual point of view the 3D videos are an interesting addition. However, from the perspective of a taxonomist, the videos are of less use since they need to be constantly played back and forth and stopped in-between to examine morphological structures. We believe that the 3D surface models in PLY format provided in our cybertype datasets provide all necessary visual data and offer much more usability since they can be freely downloaded and used with numerous types of software for multiple applications in 3D.

Overall, the relatively high quality of the 3D models and the open availability of the cybertype datasets allow taxonomists, parataxonomists, or ecologists detailed and comprehensive examinations of Afrotropical *Zasphinctus*, hence alleviating the necessity to organise multiple loans or having to visit several natural history collections. By providing our 3D models available for download on online data repositories and as interactive models on Sketchfab we also target a wider audience. Interested users can download the models and open them in a free 3D model viewer software, such as Meshlab, on a regular computer with moderate computational power and perform any subsequent visualisation or manipulation in 3D. However, the 3D models on Sketchfab require much less computation and even be viewed on a mobile phone, thus targeting a different group of users that either do not have computational means or are away from such resources, for example in the field.

### ﻿Conclusions

Despite the fact that Afrotropical *Zasphinctus* were revised recently, herein we double the number of species from five to ten. Our new data is based on few recent collections in the Afrotropical region plus extra research in historical natural history collections in Europe. Thus, we would like to highlight the need for larger scale sampling efforts throughout sub-Saharan Africa, which will almost certainly yield additional new species of *Zasphinctus*, as well as from many other ant groups.

This study further emphasises the prospects of in-depth comparative morphology analyses for insect taxonomy that are based on our integrative approach of traditional examination of physical specimens under the light microscopy and the virtual study of 3D models on the computer screen. Our newly proposed taxonomic system is based on a wealth of morphological characters of high diagnostic value, which we would not have been able to study and visualise with traditional means alone. Moreover, taking into consideration the absence of a molecular phylogeny for Afrotropical *Zasphinctus*, our taxonomic revision with the species hypotheses proposed represents a great foundation for future molecular studies, for both the Afrotropics and globally.

## Supplementary Material

XML Treatment for Zasphinctus lumumbai

XML Treatment for Zasphinctus obamai

XML Treatment for Zasphinctus wilsoni

XML Treatment for Zasphinctus aprilia

XML Treatment for Zasphinctus kouakoui

XML Treatment for Zasphinctus lolae

XML Treatment for Zasphinctus ndouri

XML Treatment for Zasphinctus sarowiwai

## ﻿Additional information

### Author ORCIDs

Francisco Hita Garcia https://orcid.org/0000-0003-4709-3083

Kiko Gómez https://orcid.org/0000-0003-4748-157X

Roberto A. Keller https://orcid.org/0000-0003-2751-9761

Bernhard Schurian https://orcid.org/0000-0001-6855-9941

Evan P. Economo https://orcid.org/0000-0001-7402-0432

### Data availability

All of the data that support the findings of this study are available in the main text or Supplementary Information.

## Supplementary materials

Supplementary material 1 List of consistent morphological differences separating the *Zasphinctusobamai* group from the *Zasphinctussarowiwai* group Data type xlsx https://binary.pensoft.net/file/1213444This dataset is made available under the Open Database License (http://opendatacommons.org/licenses/odbl/1.0/). The Open Database License (ODbL) is a license agreement intended to allow users to freely share, modify, and use this Dataset while maintaining this same freedom for others, provided that the original source and author(s) are credited.Author: Francisco Hita Garcia

Supplementary material 2 Character matrix showing all diagnostic characters used for worker-based species delimitation system of Afrotropical *Zasphinctus* Data type xlsx https://binary.pensoft.net/file/1213445This dataset is made available under the Open Database License (http://opendatacommons.org/licenses/odbl/1.0/). The Open Database License (ODbL) is a license agreement intended to allow users to freely share, modify, and use this Dataset while maintaining this same freedom for others, provided that the original source and author(s) are credited.Author: Francisco Hita Garcia

Supplementary material 3 Measurement raw data used for the comparative data of measurements and indices of Afrotropical *Zasphinctus* (including unique specimen ID, species ID, and collection locality for each specimen) Data type xlsx https://binary.pensoft.net/file/1213446This dataset is made available under the Open Database License (http://opendatacommons.org/licenses/odbl/1.0/). The Open Database License (ODbL) is a license agreement intended to allow users to freely share, modify, and use this Dataset while maintaining this same freedom for others, provided that the original source and author(s) are credited.Author: Francisco Hita Garcia

## References

[B1] Agavekar G Hita Garcia F Economo EP (2017) Taxonomic overview of the hyperdiverse ant genus *Tetramorium* Mayr (Hymenoptera, Formicidae) in India with descriptions and X-ray microtomography of two new species from the Andaman Islands. PeerJ 5: e3800. 10.7717/peerj.3800 PMC5610556 28948101

[B2] Akkari N Enghoff H Metscher BD (2015) A new dimension in documenting new species: high-detail imaging for myriapod taxonomy and first 3D cybertype of a new millipede species (Diplopoda, Julida, Julidae). PLoS ONE 10: e0135243. 10.1371/journal.pone.0135243 PMC4550252 26309113

[B3] Akkari N Ganske A-S Komerički A Metscher B (2018) New avatars for Myriapods: complete 3D morphology of type specimens transcends conventional species description (Myriapoda, Chilopoda). PLoS ONE 13: e0200158. 10.1371/journal.pone.0200158 PMC6029791 29969504

[B4] Bolton B (1976) The ant tribe Tetramoriini (Hymenoptera: Formicidae). Constituent genera, review of smaller genera and revision of *Triglyphothrix* Forel. Bulletin of the British Museum (Natural History), Entomology 34: 281–379.

[B5] Bolton B (1980) The ant tribe Tetramoriini (Hymenoptera: Formicidae). The genus *Tetramorium* Mayr in the Ethiopian zoogeographical region. Bulletin of the British Museum (Natural History), Entomology 40: 193–384.

[B6] Bolton B (1985) The ant genus *Triglyphothrix* Forel a synonym of *Tetramorium* Mayr (Hymenoptera: Formicidae). Journal of Natural History 19: 243–248. 10.1080/00222938500770191

[B7] Bolton B (1987) A review of the *Solenopsis* genus-group and revision of Afrotropical *Monomorium* Mayr (Hymenoptera: Formicidae). Bulletin of the British Museum (Natural History). Entomology 54: 263–452.

[B8] Bolton B (1990) Abdominal characters and status of the cerapachyine ants (Hymenoptera, Formicidae). Journal of Natural History 24: 53–68. 10.1080/00222939000770051

[B9] Bolton B (2024) An online catalog of the ants of the world. http://antcat.org [accessed 12 June 2024]

[B10] Borowiec ML (2016) Generic revision of the ant subfamily Dorylinae (Hymenoptera, Formicidae). Zookeys 608: 1–280. 10.3897/zookeys.608.9427 PMC4982377 27559303

[B11] Brown WL (1975) Contributions toward a reclassification of the Formicidae. V. Ponerinae, tribes Platythyreini, Cerapachyini, Cylindromyrmecini, Acanthostichini, and Aenictogitini. Search Agriculture Entomology (Ithaca) 5: 1–115. 10.5281/zenodo.26999

[B12] Buschinger A Peeters C Crozier RH (1989) Life-pattern studies on an Australian *Sphinctomyrmex* (Formicidae: Ponerinae; Cerapachyini): functional polygyny, brood periodicity and raiding behavior. Psyche 96: 287–300. 10.1155/1989/13614

[B13] Carbayo F Francoy TM Giribet G (2016) Non-destructive imaging to describe a new species of Obama land planarian (Platyhelminthes, Tricladida). Zoologica Scripta 45: 566–578. 10.1111/zsc.12175

[B14] Cignoni P Callieri M Corsini M Dellepiane M Ganovelli F Ranzuglia G (2008) MeshLab: an Open-Source Mesh Processing Tool. Sixth Eurographics Italian Chapter Conference, Salerno, Italy. 129–136.

[B15] Csősz S Fisher BL (2016) Toward objective, morphology-based taxonomy: a case study on the Malagasy *Nesomyrmexsikorai* species group (Hymenoptera: Formicidae). PLoS ONE 11: e0152454. 10.1371/journal.pone.0152454 PMC4838242 27097219

[B16] De Queiroz K (2007) Species concepts and species delimitation. Systematic Biology 56: 879–886. 10.1080/10635150701701083 18027281

[B17] Englund M Lee KM Staude H Duplouy A Hausmann A Laiho E Söderholm M Sihvonen P (2024) 130 years from discovery to description: micro-CT scanning applied to construct the integrative taxonomy of a forgotten moth from Southern Africa (Lepidoptera: Geometridae). Systematic Entomology 49: 507–525. 10.1111/syen.12627

[B18] Evenhuis NL (2024) The insect and spider collections of the world website. http://hbs.bishopmuseum.org/codens [accessed 12 June 2024]

[B19] Faulwetter S Vasileiadou A Kouratoras M Dailianis T Arvanitidis C (2013) Micro-computed tomography: Introducing new dimensions to taxonomy. ZooKeys 263: 1–45. 10.3897/zookeys.263.4261 PMC3591762 23653515

[B20] Fernández R Kvist S Lenihan J Giribet G Ziegler A (2014) Sine systemate chaos? A versatile tool for earthworm taxonomy: Non-destructive imaging of freshly fixed and museum specimens using micro-computed tomography. PLoS ONE 9: e96617. 10.1371/journal.pone.0096617 PMC4023944 24837238

[B21] Fischer G Sarnat E Economo EP (2016) Revision and microtomography of the *Pheidoleknowlesi* group, an endemic ant radiation in Fiji (Hymenoptera, Formicidae, Myrmicinae). PLoS ONE 11: e0158544. 10.1371/journal.pone.0158544 PMC4963041 27462877

[B22] Fisher BL (2009) Chapter 2 Biogeography. In: Lach L, Parr C, Abbott K (Eds) Ant Ecology Oxford Academic, Oxford, 18–37. 10.1093/acprof:oso/9780199544639.003.0002

[B23] Friedrich F Matsumura Y Pohl H Bai M Hörnschemeyer T Beutel RG (2014) Insect morphology in the age of phylogenomics: innovative techniques and its future role in systematics. Entomological Science 17: 1–24. 10.1111/ens.12053

[B24] Giribet G (2010) A new dimension in combining data? The use of morphology and phylogenomic data in metazoan systematics. Acta Zoologica 91: 11–9. 10.1111/j.1463-6395.2009.00420.x

[B25] Gómez K (2022) A revision of the Afrotropical species of the Dorylinae ant genus *Aenictus* (Hymenoptera: Formicidae) based on the worker caste. Belgian Journal of Entomology 124: 1–86.

[B26] Gómez K Kouakou LMM Fischer G Hita Garcia F Katzke J Economo EP (2022) *Pheidoleklaman* sp. nov.: a new addition from Ivory Coast to the Afrotropical *pulchella* species group (Hymenoptera, Formicidae, Myrmicinae). ZooKeys 1104: 129–157 10.3897/zookeys.1104.81562 36761928 PMC9848783

[B27] Guénard B Weiser M Dunn R (2012) Global models of ant diversity suggest regions where new discoveries are most likely are under disproportionate deforestation threat. Proceedings of the National Academy of Sciences of the United States of America 109: 7368–7373. 10.1073/pnas.1113867109 22529355 PMC3358832

[B28] Harris RA (1979) A glossary of surface sculpturing. Occasional Papers in Entomology, State of California Department of Food and Agriculture 28: 1–31. 10.5281/zenodo.26215

[B29] Hebert PDN Gregory TR (2005) The promise of DNA barcoding for taxonomy. Systematic Biology 54, 852–859. 10.1080/10635150500354886 16243770

[B30] Hita Garcia F Fischer G Peters MK Snelling RR Wägele JW (2009) A preliminary checklist of the ants (Hymenoptera: Formicidae) of Kakamega Forest (Kenya). Journal of East African Natural History 98: 147–165. 10.2982/028.098.0201

[B31] Hita Garcia F Wiesel E Fischer G (2013) The ants of Kenya (Hymenoptera: Formicidae) - Faunal overview, first species checklist, bibliography, accounts for all genera, and discussion on taxonomy and zoogeography. Journal of East African Natural History 101: 127–222. 10.2982/028.101.0201

[B32] Hita Garcia F Fischer G Liu C Audisio TL Economo EP (2017a) Next-generation morphological character discovery and evaluation: an X-ray micro-CT enhanced revision of the ant genus *Zasphinctus* Wheeler (Hymenoptera, Formicidae, Dorylinae) in the Afrotropics. ZooKeys 693: 33–93. 10.3897/zookeys.693.13012 PMC5777420 29362522

[B33] Hita Garcia F Fischer G Liu C Audisio TL Alpert GD Fisher BL Economo EP (2017b) X-ray microtomography for ant taxonomy: an exploration and case study with two new *Terataner* (Hymenoptera, Formicidae, Myrmicinae) species from Madagascar. PLoS ONE 12: e0172641. 10.1371/journal.pone.0172641 PMC5362212 28328931

[B34] Hita Garcia F Fischer G Liu C Audisio TL Economo EP (2017c) Data from: Next-generation morphological character discovery and evaluation: an X-ray micro-CT enhanced revision of the ant genus *Zasphinctus* Wheeler (Hymenoptera, Formicidae, Dorylinae) in the Afrotropics [Dataset]. Dryad Digital Repository. 10.5061/dryad.4s3v1 PMC5777420 29362522

[B35] Hita-Garcia F Lieberman Z Audisio TL Liu C Economo EP (2019) Revision of the highly specialized ant genus *Discothyrea* (Hymenoptera: Formicidae) in the Afrotropics with X-ray microtomography and 3D cybertaxonomy. Insect Systematics and Diversity 3(5): 1–84. 10.1093/isd/ixz015

[B36] Jaitrong W Yamane S (2011) Synopsis of *Aenictus* species groups and revision of the *A.currax* and *A.laeviceps* groups in the eastern Oriental, Indo-Australian, and Australasian regions (Hymenoptera: Formicidae: Aenictinae). Zootaxa 3128: 1–46. 10.11646/zootaxa.3128.1.1

[B37] Jaitrong W Wiwatwitaya D Sakchoowong W (2016) Review of the Thai species of the genus *Sphinctomyrmex* Mayr, 1866 (Hymenoptera: Formicidae, Dorylinae), with description of a new species. Far Eastern Entomologist 305: 1–9. 10.5281/zenodo.46404

[B38] Janicki J Narula N Ziegler M Guénard B Economo EP (2016) Visualizing and interacting with large-volume biodiversity data using client-server web-mapping applications: The design and implementation of antmaps.org. Ecological Informatics 32: 185–193. 10.1016/j.ecoinf.2016.02.006

[B39] Jimoh BO Gómez K Kemabonta KA Wakanjuola WA Phiri EE Mothapo PN (2024) A checklist of Nigerian ants (Hymenoptera, Formicidae): a review, new records and exotic species. Biodiversity Data Journal 12: e99555. 10.3897/BDJ.12.e99555 PMC10848766 38328409

[B40] Kass JM Guénard B Dudley KL Jenkins CN Azuma F Fisher BL Parr CL Gibb H Longino JT Ward PS Chao A Lubertazzi D Weiser M Jetz W Guralnick R Blatrix R Lauriers JD Donoso DA Georgiadis C Gomez K Hawkes PG Johnson RA Lattke JE MacGown JA Mackay W Robson S Sanders NJ Dunn RR Economo EP (2022) The global distribution of known and undiscovered ant biodiversity. Science Advances 8: eabp9908. 10.1126/sciadv.abp9908 PMC9348798 35921404

[B41] Keller RA (2011) A phylogenetic analysis of ant morphology (Hymenoptera, Formicidae) with special reference to the poneromorph subfamilies. Bulletin of the American Museum of Natural History 355: 1–90. 10.1206/355.1

[B42] Michalik P Ramírez MJ (2013) First description of the male of *Thaidachepu* Platnick, 1987 (Araneae, Austrochilidae) with micro-computed tomography of the palpal organ. ZooKeys 352: 117–125. 10.3897/zookeys.352.6021 PMC3837400 24294094

[B43] Miller SE (2007) DNA barcoding and the renaissance of taxonomy. PNAS 104: 4775–4776. 10.1073/pnas.0700466104 17363473 PMC1829212

[B44] Moraes SS Söderholm MS Aguiar TMC Freitas AVL Sihvonen P (2023) Micro-CT imaging in species description: exploring beyond sclerotized structures in lichen moths (Lepidoptera: Erebidae, Arctiinae, Lithosiini). PeerJ 11: e15505. 10.7717/peerj.15505 PMC10351509 37465151

[B45] Reizebos EP Vooren AP Guillaumet JL [Eds] (1994) The Taï National Park, Côte d’Ivoire. I: Synthesis of knowledge (Report). La Fondation Tropenbos, Horaplantsoen. [ISBN 90-5113-020-1]

[B46] Robertson HG (2000) Afrotropical ants (Hymenoptera: Formicidae): taxonomic progress and estimation of species richness. Journal of Hymenoptera Research 9: 71–84.

[B47] Sadasivan K Kripakaran M (2022) First record of *Proceratium* Roger, 1863, *Zasphinctus* Wheeler, 1918, and *Vollenhovia* Mayr, 1865 (Hymenoptera: Formicidae) from the Western Ghats of peninsular India, description of three new species, and implications for Indian biogeography. Journal of Threatened Taxa 14: 21368–21387. 10.11609/jott.7682.14.7.21368-21387

[B48] Sarnat E Fischer G Economo EP (2016) Inordinate spinescence: taxonomic revision and microtomography of the *Pheidolecervicornis* species group (Hymenoptera, Formicidae). PLoS ONE 11: e0156709. 10.1371/journal.pone.0156709 PMC4963106 27463644

[B49] Sarnat EM Hita Garcia F Dudley K Liu C Fischer G Economo EP (2019) Ready species one: exploring the use of augmented reality to enhance systematic biology with a revision of Fijian *Strumigenys* (Hymenoptera: Formicidae). Insect Systematics and Diversity 3(6): 1–43. 10.1093/isd/ixz005

[B50] Schlick-Steiner BC Steiner FM Seifert B Stauffer C Christian E Crozier RH (2010) Integrative taxonomy: a multisource approach to exploring biodiversity. Annual Review of Entomology 55: 421–438. 10.1146/annurev-ento-112408-085432 19737081

[B51] Sharaf MR Aldawood AS Economo EP Wachkoo AA Hita Garcia F (2019) Taxonomy of Arabian *Temnothorax* Mayr (Formicidae: Myrmicinae) with description of a new species enhanced by x-ray microtomography. Scientific Reports 9: 11009 10.1038/s41598-019-47260-y PMC6662808 31358795

[B52] Simonsen TJ Kitching IJ (2014) Virtual dissections through micro-CT scanning: a method for non-destructive genitalia ‘dissections’ of valuable Lepidoptera material. Systematic Entomology 39: 606–618. 10.1111/syen.12067

[B53] Staab M Hita Garcia F Liu C Xu Z-H Economo EP (2018) Systematics of the ant genus *Proceratium* Roger (Hymenoptera, Formicidae, Proceratiinae) in China – with descriptions of three new species based on micro-CT enhanced next-generation-morphology. ZooKeys 770: 137–192. 10.3897/zookeys.770.24908 PMC6041363 30002593

[B54] Stoev P Komerički A Akkari N Liu S Zhou X Weigand AM Hostens J Hunter CI Edmunds SC Porco D Zapparoli M Georgiev T Mietchen D Roberts D Faulwetter S Smith V Penev L (2013) *Eupolybothruscavernicolus* Komerički & Stoev sp. n. (Chilopoda: Lithobiomorpha: Lithobiidae): the first eukaryotic species description combining transcriptomic, DNA barcoding and micro-CT imaging data. Biodiversity Data Journal 1: e1013. 10.3897/BDJ.1.e1013 PMC3964625 24723752

[B55] Ward PS (2000) Broad-scale patterns of diversity in leaf litter ant communities. In: Agosti D Majer J Alonso L Schultz TR (Eds) Ants: standard methods for measuring and monitoring biodiversity.Smithsonian Institution Press, Washington, DC, 99–121. https://zenodo.org/record/11736

[B56] Ward PS (2011) Integrating molecular phylogenetic results into ant taxonomy (Hymenoptera: Formicidae). Myrmecological News 15: 21–9. 10.25849/myrmecol.news_015:021

[B57] Wilson EO (1958) Observations on the behavior of the cerapachyine ants. Insectes Sociaux 5: 129–140. 10.1007/BF02222432

[B58] Wilson EO (1964) The true army ants of the Indo-Australian area (Hymenoptera: Formicidae: Dorylinae). Pacific Insects 6: 427–483.

